# Regulatory mechanisms of fatty acids biosynthesis in *Armeniaca sibirica* seed kernel oil at different developmental stages

**DOI:** 10.7717/peerj.14125

**Published:** 2022-10-04

**Authors:** Yueliang Wu, Wenya Gao, Xinli Li, Shilin Sun, Jian Xu, Xiaoqiong Shi, Huiyan Guo

**Affiliations:** 1 College of Forestry, Shenyang Agricultural University, Shenyang, Liaoning, China; 2 The Key Laboratory of Forest Tree Genetics, Breeding and Cultivation of Liaoning Province, Shenyang Agricultural University, Shenyang, Liaoning, China

**Keywords:** *Armeniaca sibirica*, Seed kernel, Fatty acid composition, Biosynthesis, Key enzymes, Transcription factors, Long non-coding RNAs, Regulatory mechanism

## Abstract

**Background:**

*Armeniaca sibirica* seed kernel oil is rich in oleic acid and linoleic acid, thus holding potential value as a source of high-quality edible oils. However, some regulatory factors involved in fatty acids accumulation in* A. sibirica* seed kernels remain largely elusive. Thus, the aim of this study was to elucidate the regulatory mechanisms underlying fatty acids biosynthesis in *A. sibirica* developing seed kernels.

**Methods:**

Seed kernels from six plants from a single *A. sibirica* clone were taken at five different developmental stages (days 30, 41, 52, 63, and 73 after anthesis). Fatty acid composition in seed kernel oil was determined by gas chromatography-mass spectrometry (GC-MS). In addition, transcriptome analysis was conducted using second-generation sequencing (SGS) and single-molecule real-time sequencing (SMRT).

**Results:**

Rapid accumulation of fatty acids occurred throughout the different stages of seed kernels development, with oleic acid and linoleic acid as the main fatty acids. A total of 10,024, 9,803, 6,004, 6,719 and 9,688 unigenes were matched in the Nt, Nr, KOG, GO and KEGG databases, respectively. In the category lipid metabolism, 228 differentially expressed genes (DEGs) were annotated into 13 KEGG pathways. Specific unigenes encoding 12 key enzymes related to fatty acids biosynthesis were determined. Co-expression network analysis identified 11 transcription factors (TFs) and 13 long non-coding RNAs (lncRNAs) which putatively participate in the regulation of fatty acid biosynthesis. This study provides insights into the molecular regulatory mechanisms of fatty acids biosynthesis in *A. sibirica* developing seed kernels, and enabled the identification of novel candidate factors for future improvement of the production and quality of seed kernel oil by breeding.

## Introduction

Plants can biosynthesize and accumulate oils in seeds as a source of carbon and energy for seedling development. Plant seed oils also have various industrial applications; for example, cosmetics, lubricants, surfactants, and renewable biodiesel feedstocks. In addition, plant oils are important components of the human diet in the form of edible oils. It is estimated that the global demand for plant seed oils will double by 2030, which will require an increase in plant oil production to meet the growing demand (Chapman & Ohlrogge, 2012).

Plant seed oils are mainly composed of higher fatty acids (FAs), monounsaturated fatty acids (MUFAs), and polyunsaturated fatty acids (PUFAs), including very-long-chain fatty acids (VLCFAs), oleic acid, linoleic acid, and linolenic acid, which have valuable industrial applications and/or can be used as food oils. VLCFAs, such as erucic and nervonic acids, are commonly used as industrial feedstocks (Li et al., 2012; Marillia et al., 2014). VLCFAs can be synthesized from acyl-CoA substrates by the FA elongase (FAE) complex; the first condensation reaction in the FAE complex is catalyzed by the 3-ketoacyl-CoA synthase (KCS) and is a rate-limiting step for the accumulation of VLCFAs (Millar & Kunst, 1997). It has been shown that the production of VLCFAs in *Arabidopsis* seeds increased due to the synergetic increase in *FAE1* (KCS18) expression and substrate availability (Ma et al., 2021).

MUFAs (*e.g.*, oleic acid) are more stable for storage of food oils than PUFAs, being also healthier and more easily absorbed by the human body compared to saturated fatty acids (SFAs) (Huang et al., 2016). Therefore, increasing the content of oleic acid has become a key attribute to be achieved in edible oil plant breeding. Fatty acid desaturase FAD2 degrades oleic acid to generate linoleic acid with two unsaturated bonds, whereas fatty acid desaturase FAD3 degrades linoleic acid to generate linolenic acid with three unsaturated bonds. Thus, these two enzymes play a key role in determining fatty acid unsaturation (Dar et al., 2017; Choudhary & Mishra, 2021). In a recent study, two novel mutant BnFAD2 alleles were shown to increase the content of oleic acid in *Brassica napus* seed oil (Fu et al., 2021). In *Hexaploid camelina*, silencing two CsFAD2 homologs led to an increase in MUFAs content (Lee et al., 2021). In contrast, high content of erucic acid (C22:1) in edible oils results in loss of nutritional quality (Shyam et al., 2021). Thus, another important attribute to consider in edible oil breeding is to achieve low contents of erucic acid. It has been demonstrated that RNA-interfering transgenic rapeseeds, namely BnFAD2-Ri, BnFAE1-Ri and BnFAD2/BnFAE1-Ri, exhibited higher oleic acid and lower erucic acid contents (Shi et al., 2017). In addition, insertion and deletion (indel) mutations of FAE1 constructed with the CRISPR-Cas9 technology abolished erucic acid production, thus resulting in an edible pennycress seed oil comparable to that of canola (McGinn et al., 2019). Taken together, fatty acids (FAs) biosynthesis can be affected by the products of these genes.

Furthermore, numerous transcription factors (TFs), including WRI1, FUS3, ABI3, LEC2 and LEC1 which are important positive regulators in FAs biosynthesis (Mcglew et al., 2015). WRI1 regulates the expressions of at least 15 genes encoding several enzymes, including pyruvate dehydrogenase complex (PDC) and acetyl-CoA carboxylase (ACC), thus playing an important role in glycolysis and FAs biosynthesis (Bates, Stymne & Ohlrogge, 2013; Kong et al., 2020; Yu et al., 2021). It has been demonstrated that the overexpression of four WRI1s (three WRI1s from *Avena sativa* and one WRI1 from *Ricinus communis*) in *Arabidopsis* and tobacco bright yellow-2 (BY-2) cells led to an increase, respectively, in the content of seed oil and total FAs in cells (Yang et al., 2019). In addition, overexpression of PoWRI1 from *Paeonia ostii* in transgenic *Arabidopsis* resulted in larger seeds and a significant increase in the contents of oil and unsaturated FAs (Sun et al., 2021). Moreover, it has been found that ABI3 interacts with FUS3, LEC2 and LEC1 to form an L-AFL network during seed maturation (Baud et al., 2016). Thus, although these regulators have been identified in FAs accumulation in several oil storage tissues, the underlying mechanisms related to these regulators in different tissues may not be conserved.

*Armeniaca sibirica* belongs to the family Rosaceae, and the oil content in *A. sibirica* seed kernels is usually within the range of 40–56% (Femenia et al., 1995). The main FAs in *A. sibirica* kernels are oleic (58.3–73.4%) and linoleic acids (18.8–31.7%) (Alpaslan & Hayta, 2006), indicating the potential application value of *A. sibirica* kernel oil as a source of biodiesel and edible feedstocks. Considerable progress has been made on the understanding of oil contents, FAs composition, and usages (Wang, 2010); however, current knowledge on the regulatory mechanisms underlying FAs accumulation in *A. sibirica* seed kernels is still insufficient. In the present study, an integrated omics approach was adopted to determine the oil content, FAs composition, and the genes involved in FAs biosynthetic pathways in *A. sibirica* seed kernels at five developmental stages after anthesis. A network of FAs biosynthesis has been proposed, and the transcriptional profiles of relevant genes were summarized. Collectively, this study enabled the identification of novel candidate genes for future use in molecular breeding which could significantly improve FAs composition of *A. sibirica*.

## Materials & Methods

### Plant materials and study design

*A. sibirica* clone no.1 was grown in a clonal seed orchard at a forest tree seed breeding center in Beipiao City (42°28′N, 120°18′E), Liaoning Province, China. Anthesis of the chosen *A. sibirica* clone occurred in April 20, 2019. Seed kernels of *A. sibirica* at five different developmental stages after anthesis were collected, *i.e.*, days 30 (SI), days 41 (SII), days 52 (SIII), days 63 (SIV), and days 73 (SV) after anthesis. Six *A. sibirica* no.1 plants were chosen, and seed kernels from each plant were taken. All collected materials were frozen immediately in liquid nitrogen and stored in −80 °C.

### Analysis of FAs composition in seed kernels

FAs composition of *A. sibirica* seed kernels was determined using a previously described method (Pan et al., 2013) with some modifications. Briefly, approximately 50 mg of seeds was used in each experiment, and salicylate methyl ester was used as an internal standard. Seeds were homogenized in a superfine homogenizer (FLUKO, Frankfurt, Germany) and methylated with two mL of 1% methanolic H_2_SO_4_ at 80 °C for 30 min. Generated FA methyl esters were extracted with hexane, dried under nitrogen gas, resuspended in 1.5 mL of dichloromethane, and analyzed in an HP-INNOWax capillary column (30 m × 0.25 mm × 0.25 µm) and a flame ionization detector (FID) (Agilent Technologies, Santa Clara, CA, USA). The following parameters were used: temperature program, 50 °C for 3 min, 10 °C min^−1^ to 220 °C and hold for 3 min, 15 °C min^−1^ to 250 °C and hold for 10 min; injection volume, 1 µL in splitless mode. Helium was used as the carrier gas at a flow rate of 1.0 mL min ^−1^. FAs were identified based on FA methyl ester standards (Sigma-Aldrich, St. Louis, MO, USA). The percentage of each FA in oils was calculated based on their corresponding peak areas. Six biological replicates were performed in these experiments. ANOVA and multiple comparisons involving FAs contents at different developmental stages were performed using SPSS v.17.0 (SPSS Inc. Chicago, IL, USA).

### RNA extraction, library construction and sequencing

Total RNA of *A. sibirica* no.1 seed kernels at five different developmental stages was isolated by using an RNA extraction kit (Tiangen Biotech Co., Ltd., Beijing, China) according to the manufacturer’s instructions. Three biological replicates were performed for each developmental stage. Quality of obtained RNA was determined by agarose gel electrophoresis. The RNA was qualified and quantified in using a Nanodrop ND-1000 Spectrophotometer (Nanodrop Technologies, Wilmington, DE, USA). The enrichment of poly(A) mRNA was conducted using oligo(dT). First strand cDNA was synthesized using random hexamer primers. Buffer, dNTPs, RNase H and DNA polymerase I (Takara Bio Inc. Shiga, Japan) were added to synthesize the second strand. Obtained double-stranded cDNA fragments were screened using BluePippin (Illimina, San Diego, CA, USA), and fragments larger than 4 Kb were enriched. Large-scale PCR was performed to enrich selected fragments to obtain sufficient cDNA. Subsequently, the screened full-length cDNA was enriched for damage and end repairs, then the SMRT dumbbell-shaped linker was connected to the cDNA fragments. Then, a library with an equimolar concentration of non-selected fragments and fragments longer than 4 Kb was constructed. Exonuclease digestion was used to remove unconnected sequence at both ends of the cDNA fragments. Finally, the complete SMRT bell library was constructed by combining primers and DNA polymerase.

The constructed cDNA library was sequenced using the PacBio Sequel platform according to the effective concentration of library fragments and data output requirements. Adapter sequences, sequences with unknown bases (N), and low-quality reads were removed to obtain clean reads. Subsequently, clean reads were assembled into unigenes. The statistical power, calculated using RNASeqPower (https://doi.org/doi:10.18129/B9.bioc.RNASeqPower) was 0.9993995.

### Transcriptome annotation and pathway enrichment analysis

Unigenes were annotated by comparing the assembled transcriptome to sequences available on the following databases: NCBI Non-redundant protein sequences (Nr: diamond v0.8.36, *e*-value = 1e−5), NCBI Nucleotide sequences (Nt: NCBI blast 2.7.1+, *e*-value = 1e−5), euKaryotic Ortholog Groups (KOG: diamond v0.8.36, *e*-value = 1e−5), Gene Ontology (GO: Blast2GO v2.5, *e*-value = 1e−6), Kyoto Encyclopedia of Genes and Genomes (KEGG: diamond v0.8.36, *e*-value = 1e−5) databases.

### Identification of differentially expressed genes (DEGs)

Differential expression analysis of *A. sibirica* seed kernels across five different developmental stages was performed using DESeq package according to the previous report (Anders & Huber, 2016). *P*-values were adjusted based on the method proposed by Benjamini et al. (2001). Genes with *P*-value <0.05 and —log _2_(Fold Change)—>0 were considered as DEGs.

### Validation of transcriptome data by qRT-PCR

To validate RNA sequencing (RNA-Seq) data, total RNA samples were reverse transcribed into cDNA using the HiScript III RT SuperMix for qPCR (Vazyme Biotech Co.,Ltd. Nanjing, China). Eight differentially expressed genes (DEGs) were randomly selected. The 18SrRNA gene was used as an internal reference gene (Table S1). Primer sequences used in qRT-PCR analysis are given in Table S1. In a final reaction volume of 20 µL, each qRT-PCR reaction contained: 10 µL of ChamQ Universal SYBR qPCR Master Mix (Vazyme Biotech Co., Ltd. Nanjing, China), 2 µL of cDNA template, 1 µL of forward primer (10 µM), 1 µL of reverse primer (10 µM), and 6 µL of ddH_2_O. Amplification conditions were as follows: 95 °C for 30 s; followed by 35 cycles of 95 °C for 20 s, 55 °C for 30 s, and 72 °C for 30 s; and final extension at 60 °C for 15 s. Relative gene expression was calculated using the 2^−ΔΔCt^ method (Pfaffl, Horgan & Dempfle, 2002). Three independent experiments were performed.

## Results

### Changes in FAs composition in developing seed kernels

Metabolome analysis revealed the presence of 24 types of FAs in *A. sibirica* seed kernel oil obtained from seeds (Table S2). The contents of various saturated fatty acids (SFAs) and unsaturated fatty acids (UFAs) at different developmental stages are shown in Table S3.

**Figure 1:**
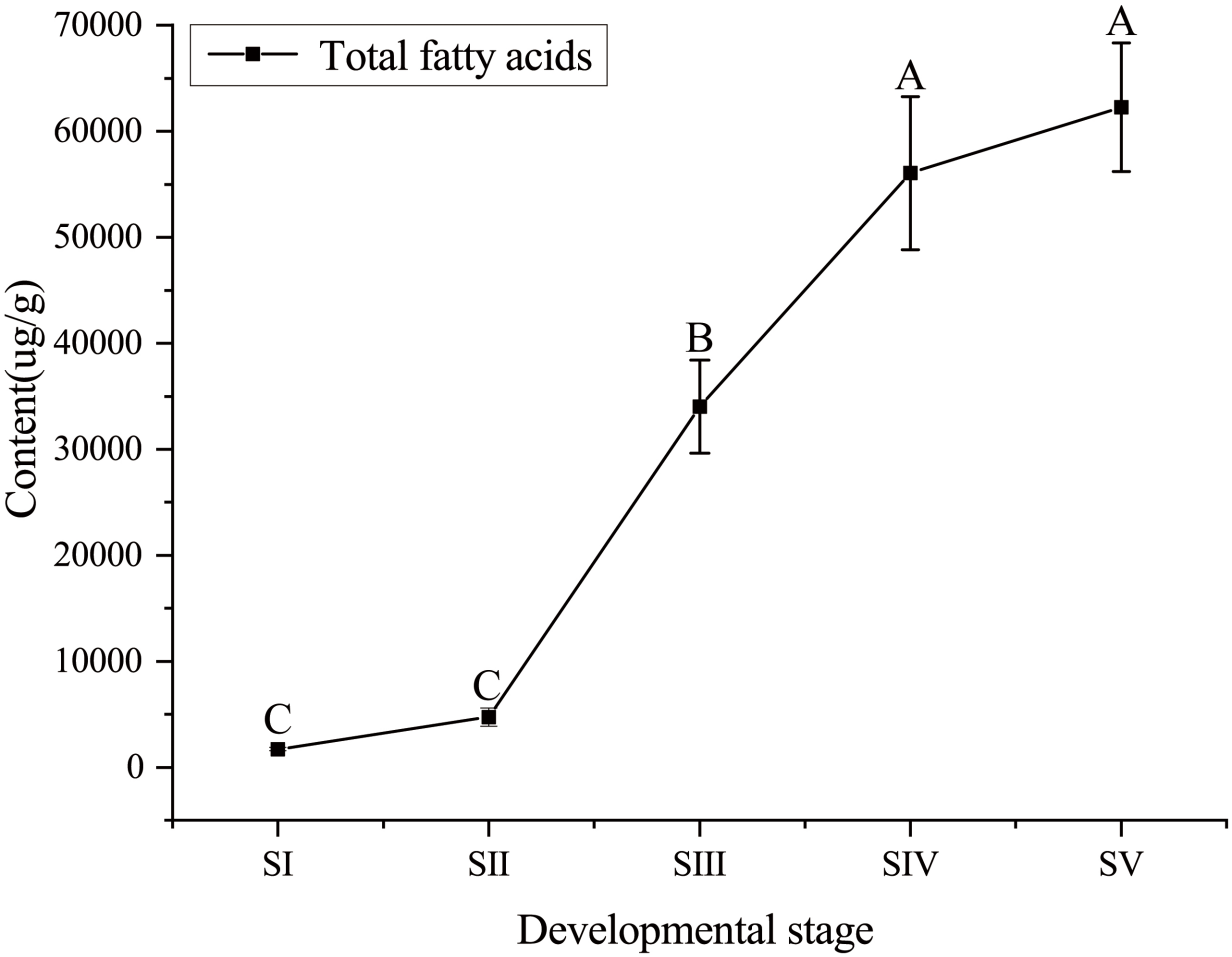
Dynamic changes in total fatty acids content in *Armeniaca sibirica* seed kernel oil obtained from seeds at five different developmental stages. Different capital letters indicate significant differences (*p* < 0.01). Six biological replicates were performed in these experiments.

**Figure 2:**
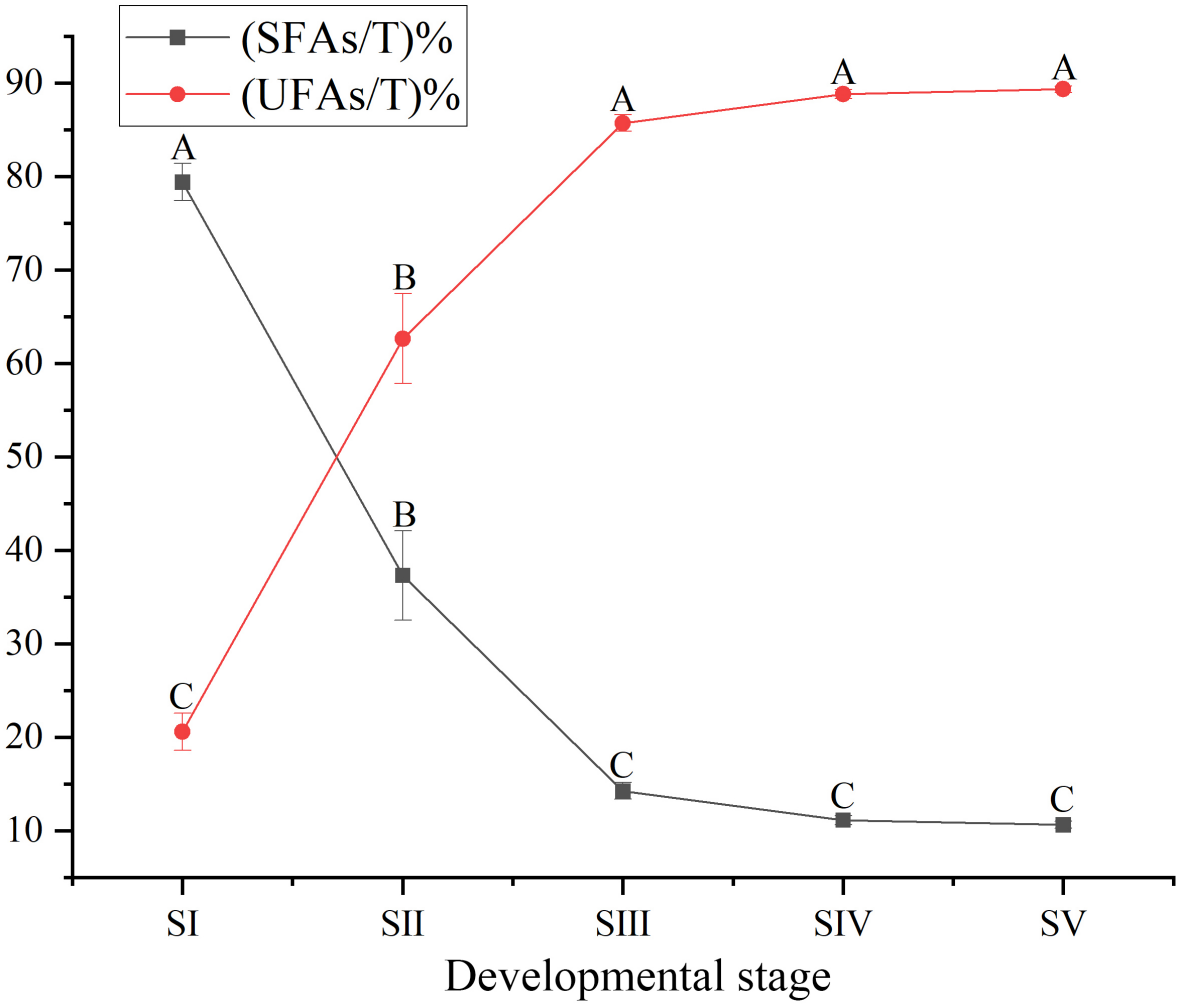
Dynamic changes in the proportions of saturated fatty acids and unsaturated fatty acids in *Armeniaca sibirica* seed kernel oils obtained from seeds at different developmental stages. Different capital letters indicate significant differences (*p* < 0.01). Six biological replicates were performed in these experiments.

The content of total FAs increased rapidly, especially from SII to SIV (Fig. 1, Table S4), with the development of seed kernels. The proportion of SFAs decreased rapidly from stages SI to SIII; conversely, the proportion of UFAs increased rapidly. At SIV and SV, changes in the proportions of SFAs and UFAs were only moderate (Fig. 2). In oils from seed kernels at the mature stage of development (SV), the content of SFAs accounted for only 10.65% of total FAs, whereas the content of UFAs accounted for 89.35% (Table S5). Thus, UFAs were the dominant FA in mature *A. sibirica* seed kernels.

**Figure 3:**
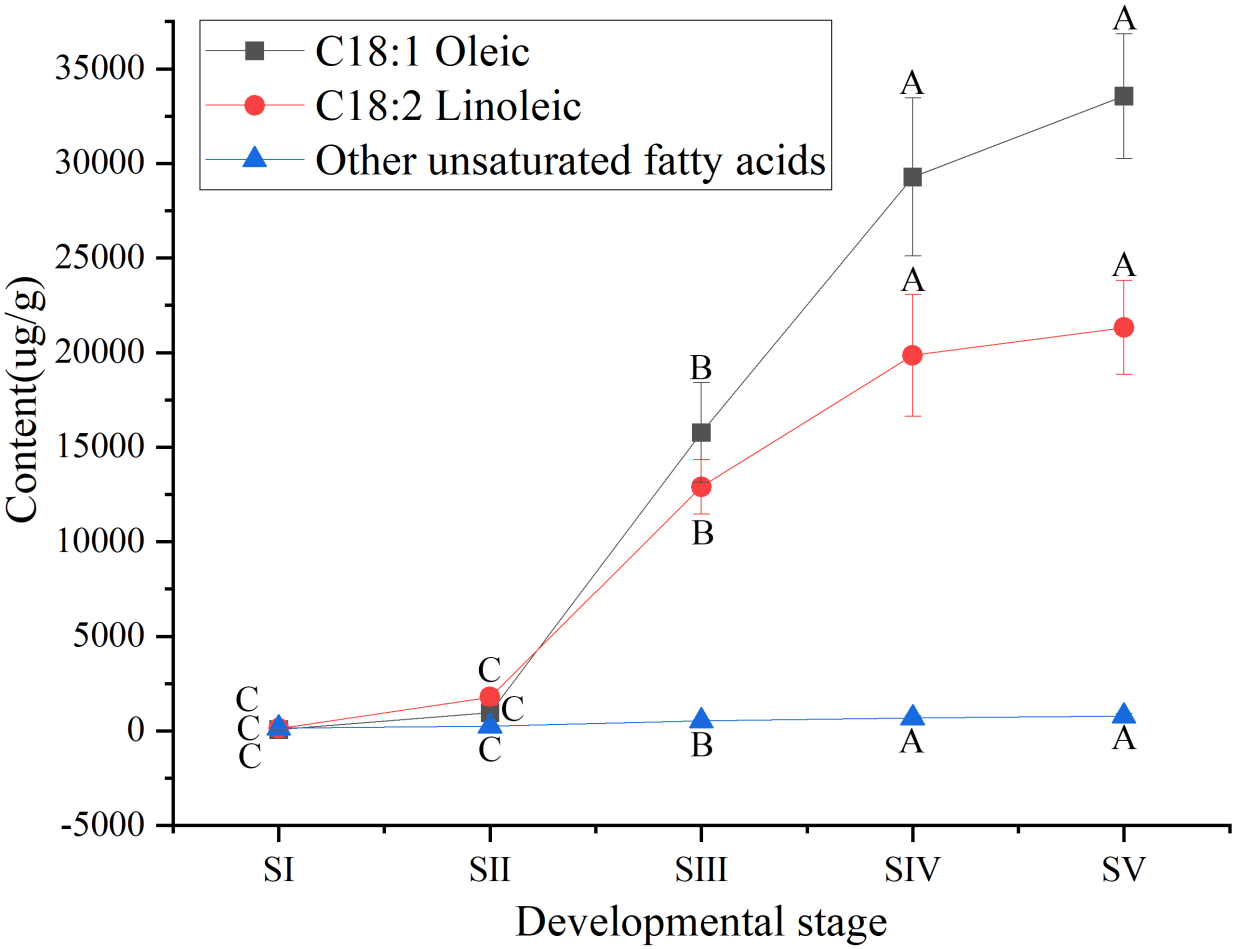
Dynamic changes in the content of main unsaturated fatty acids in *Armeniaca sibirica* seed kernels oils obtained from seeds at different developmental stages. Different capital letters indicate significant differences (*p* < 0.01). Six biological replicates were performed in these experiments.

Among UFAs (Table S3) in mature seed kernels oil (SV), the contents of oleic (C18:1) and linoleic acids (C18:2) were higher than the total amount of the other UFAs, such as palmitoleic, erucic and linolenic acids (Table S6). Biosynthesis of oleic and linoleic acids was relatively slow from stages SI to SII, and rapidly increased from stages SII to SIV, with a further increase until maturation (SV) (Fig. 3).

**Figure 4:**
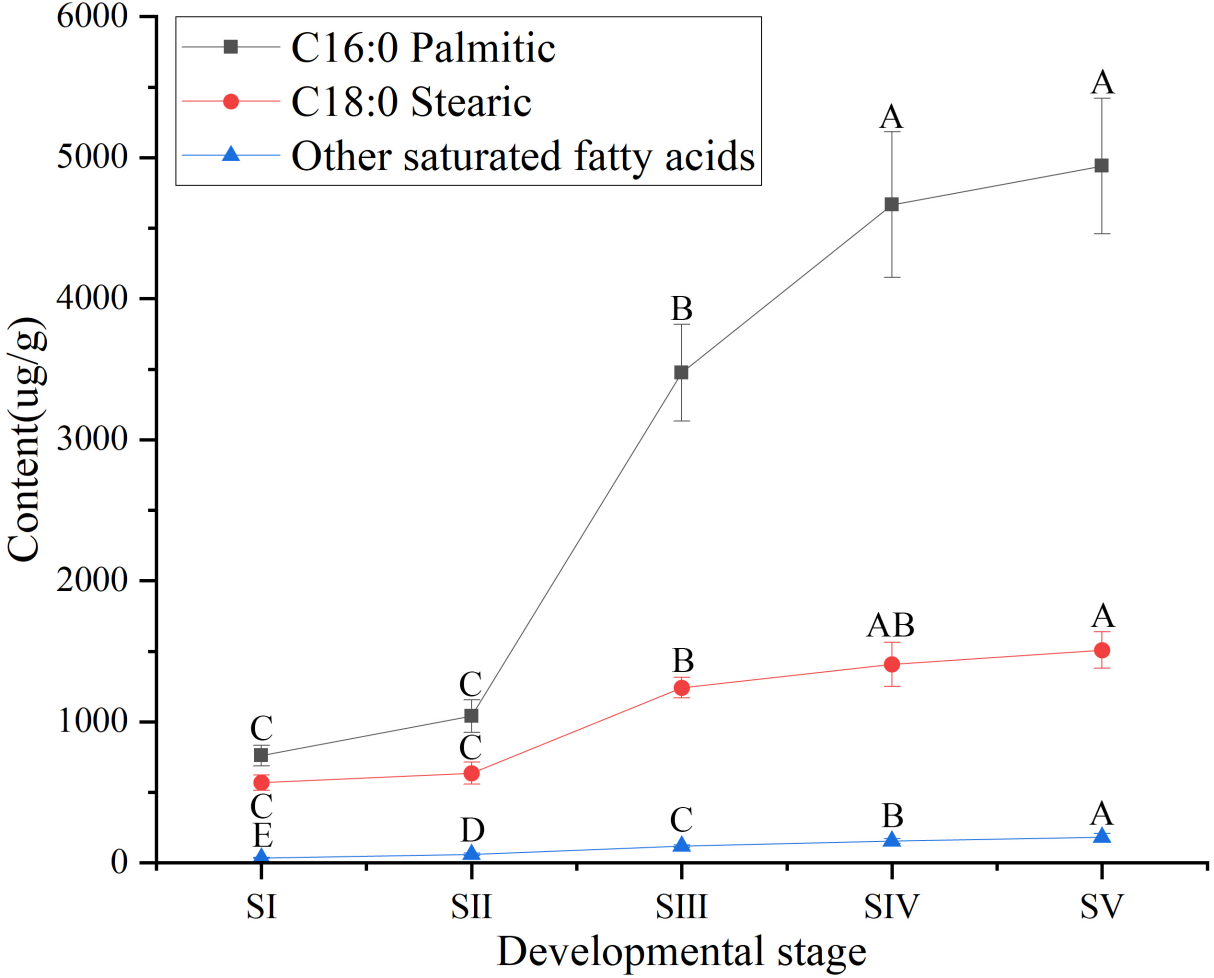
Dynamic changes in the content of main saturated fatty acids in *Armeniaca sibirica* seed kernels oil obtained from seeds at different developmental stages. Different capital letters indicate significant differences (*p* < 0.01). Six biological replicates were performed in these experiments.

Similarly, among SFAs (Table S3), the contents of palmitic (C16:0) and stearic acids (C18:0) at all stages of seed kernels development were higher than the content of the other SFAs, such as arachidic, behenic and lignoceric acids (Table S7). Although the contents of palmitic and stearic acids gradually increased throughout the development of *A. sibirica* seed kernels (Fig. 4), the contents of palmitic and stearic acids were considerably lower compared with the contents of oleic and linoleic acids (Table S3); this could be due to the fact that the precursors of both palmitic and stearic acids were directed to the synthesis of oleic and linoleic acids.

Except for oleic and linoleic acids, the contents of palmitoleic (C16:1), *α*-linolenic (C18:3N3), *γ*-linolenic (C18:3N6), erucic (C22:1) acids and other UFAs were remarkably low at the mature stage of development (SV) (Table S3). Collectively, these results indicated that oleic and linoleic acids are the main FAs in *A. sibirica* seed kernels oil. Next, we aimed to explore the key genes involved in FAs synthesis (especially of oleic and linoleic acids) and certain regulatory factors to elucidate the molecular mechanisms underlying the regulation of their biosynthesis in *A. sibirica* seed kernels oil from seeds at different developmental stages.

### Seed kernels transcriptome analysis

RNA from *A. sibirica* seed kernels at different developmental stages was extracted and used to construct five cDNA libraries separately. RNA-seq analysis indicated (Table S8) that approximately 114.97 Gb of clean data were obtained, with a total of 43,224,714 ∼61,828,066 clean reads. The clean reads rate was over 98% and the Q30 percentage representing sequencing quality was between 91.86% and 93.78%. The GC content was between 45.48% and 49.57%. The number, average length and N50 of assembled unigenes were 10,093, 1,445 bp and 1,715 bp, respectively (Table S9). Collectively, these results indicate that our data were of high continuity and quality.

### Transcriptome functional annotation

**Figure 5:**
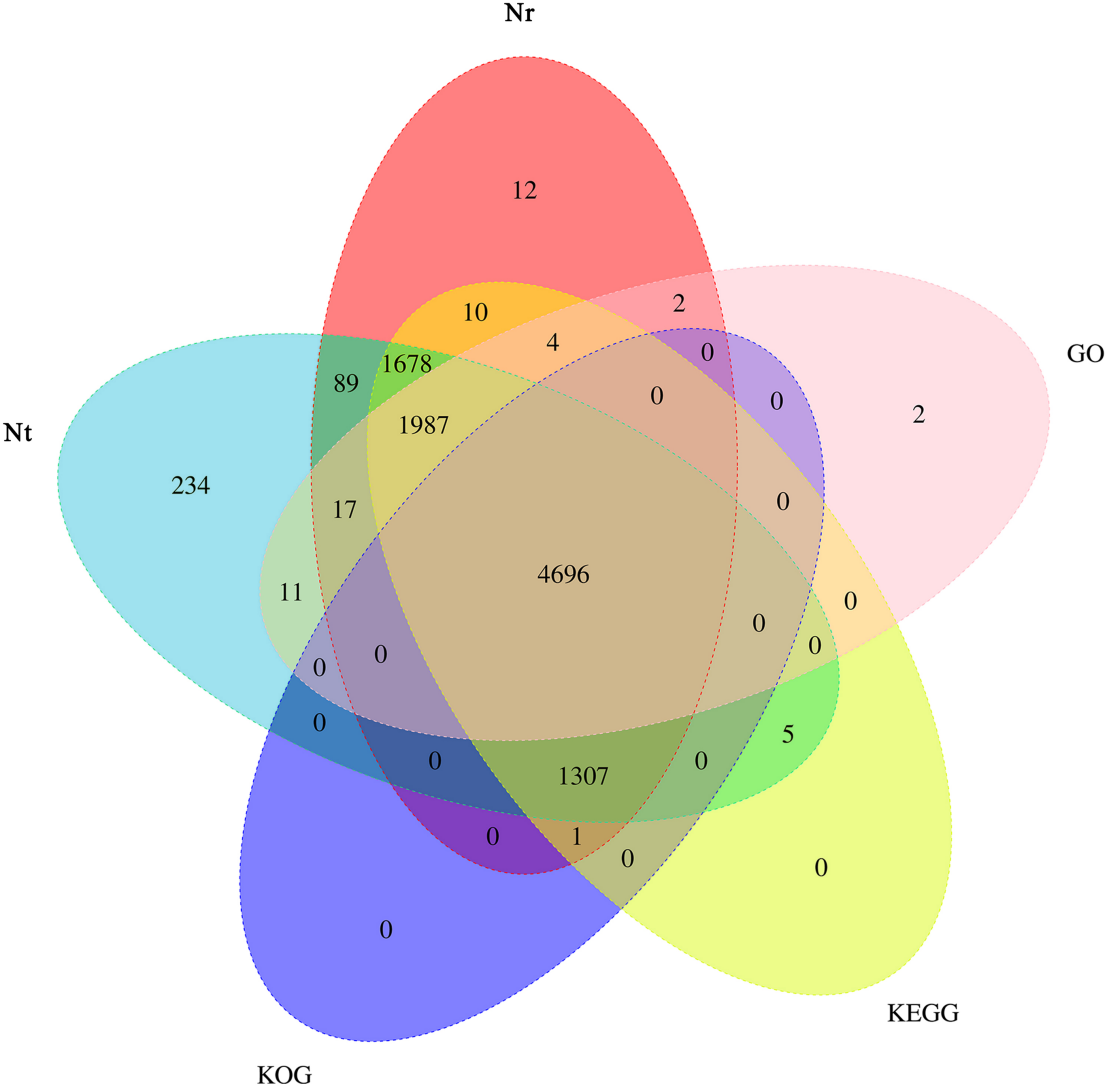
Venn diagram depicting the number of unigenes in the transcriptome of *Armeniaca sibirica* seed kernels at different developmental stages annotated in Nt, Nr, KOG, GO, KEGG.

Based on the predicted sequences in final contigs, unigenes were annotated using BLAST against the Nt, Nr, KOG, GO and KEGG databases. Briefly, 10,024, 9,803, 6,004, 6,719, and 9,688 unigenes had the most significant BLAST matches with known protein sequences available in the Nt, Nr, KOG, GO and KEGG databases, respectively. A total of 10,055 annotated transcripts were found in all the five databases, with 4,696 shared annotated transcripts, accounting for 46.70% of the total number of transcripts (Fig. 5).

**Figure 6:**
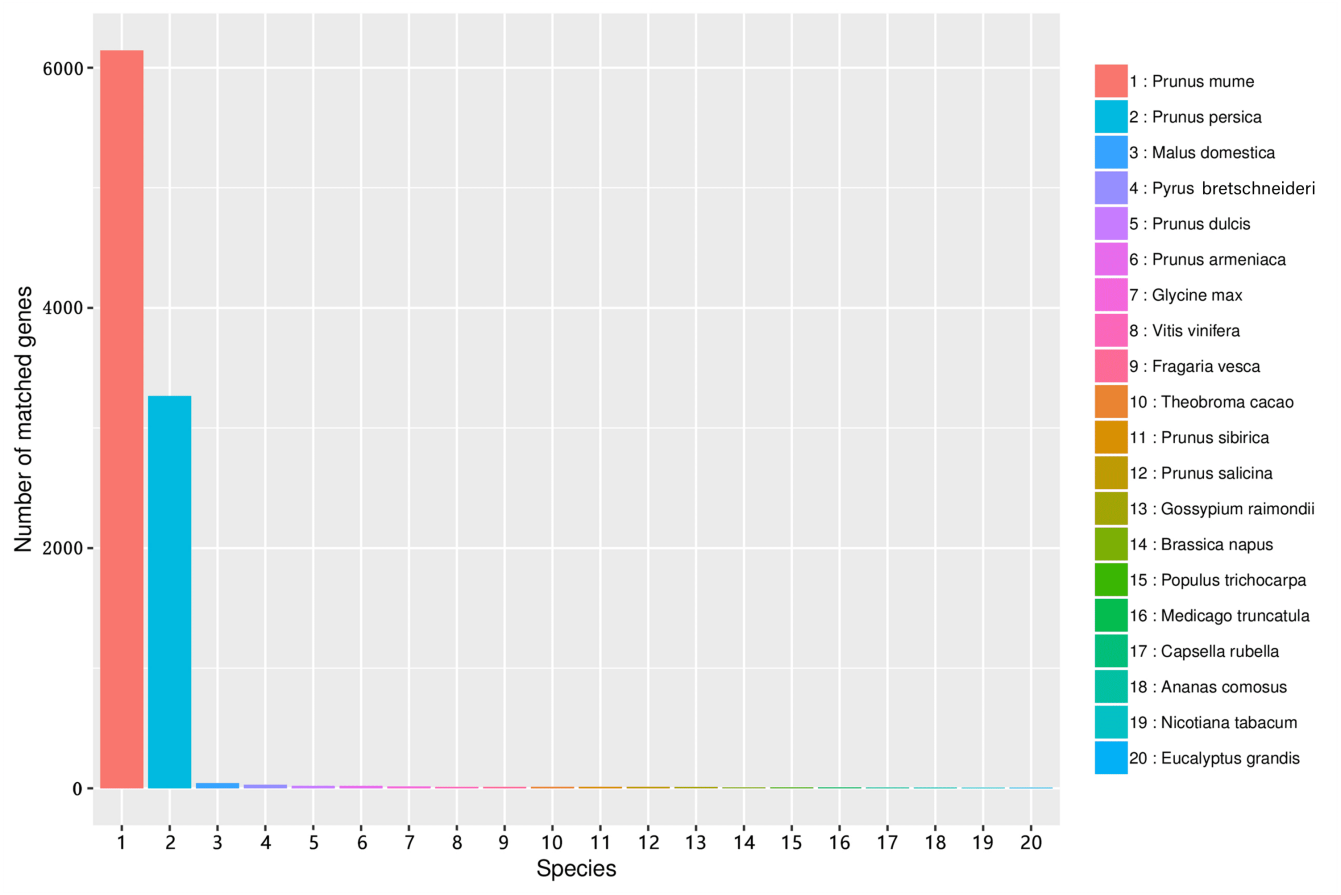
Number of unigenes in the transcriptome of *Armeniaca sibirica* matching the 20 top species using BLAST against the Nr database.

The similarity of unigene sequences between *A. sibirica* and related species was obtained by comparison using the Nr database. Figure 6 shows that the number of transcripts matched against the *Prunus mume* annotated genome was greater than 6000, accounting for more than 60% of total transcripts. In addition, the number of transcripts matched against the *Prunus persica* annotated genome was greater than 3,200, accounting for over 30% of total transcripts. In contrast, the number of unigenes of *A. sibirica* with homology to *Malus domestica*, *Pyrus bretschneideri*, and other species was low (Table S10). Collectively, these results showed that *A. sibirica* unigenes had high homology with genes of *P. mume* and *P. persica*.

**Figure 7:**
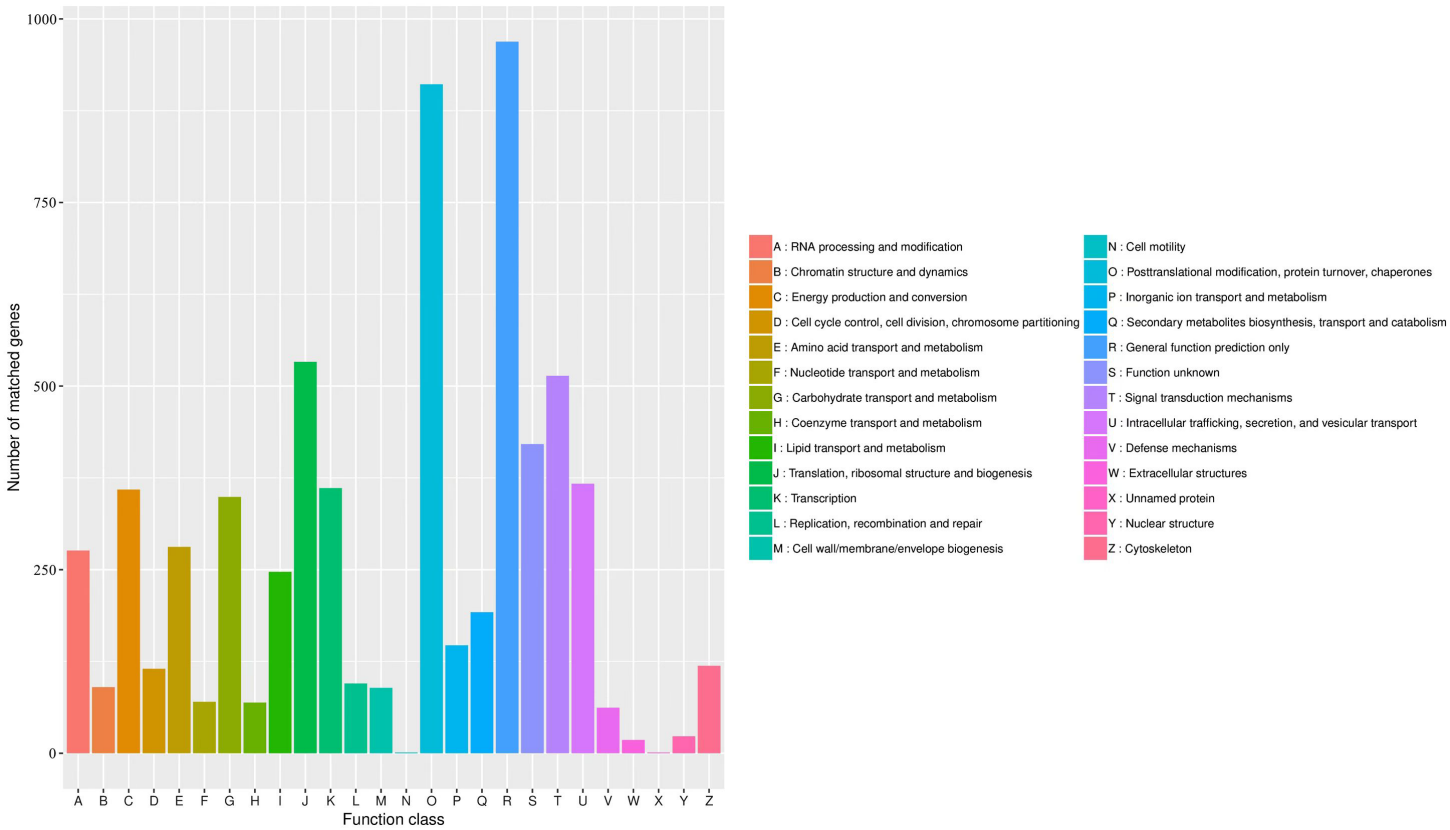
KOG annotation of *Armeniaca sibirica* transcriptome.

The KOG database was used to assign functions to the predicted unigenes in *A. sibirica*. In total, 6,004 unigenes were assigned to 26 functional categories (Fig. 7, Table S11). The largest represented group was “general function prediction only” (approximately 1,000 unigenes), which shows that a large number of unknown genes in *A. sibirica* deposited in public databases are of potential value. The second most represented group was “post-translational modification, protein turnover, chaperones” (over 900 unigenes), followed by “translation, ribosomal structure and biogenesis” (over 500 unigenes) and “signal transduction mechanisms” (over 500 unigenes). In contrast, the categories “cell motility” and “unnamed protein” only contained a few unigenes.

**Figure 8:**
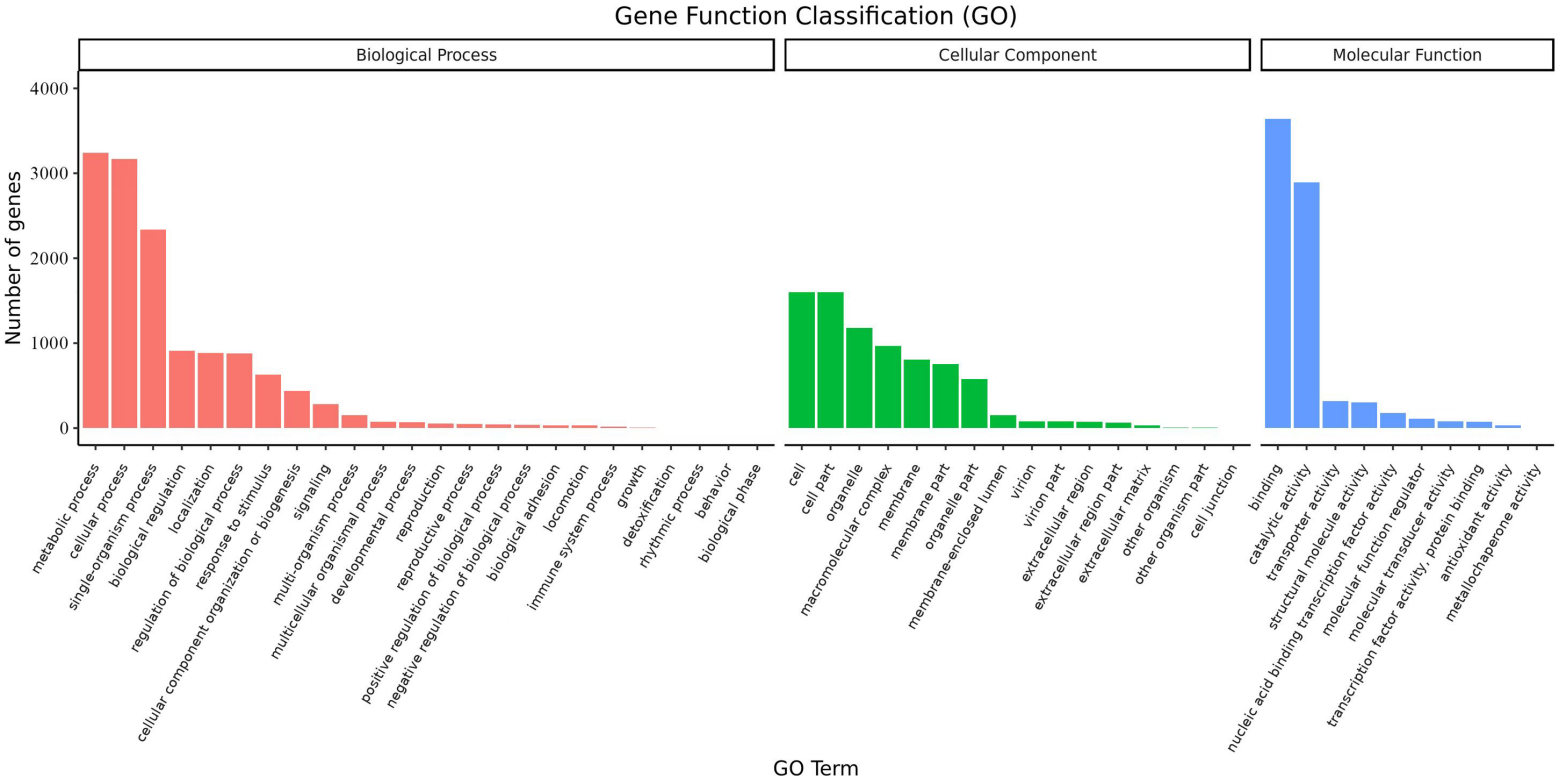
GO annotation of differentially expressed unigenes in *Armeniaca sibirica* seed kernels at different developmental stages.

Overall, 6,719 transcripts were assigned to three main GO functional categories, *i.e.*, “biological process”, “cellular component”, “molecular function”, as well as to 50 sub-categories (Fig. 8, Table S12). Transcripts assigned to the category “biological process” were further classified into 24 sub-categories; the three most represented sub-categories were “metabolic process”, “cellular process” and “single-organism process” which contained over 3,200, 3,100, and 2,300 genes, respectively. Additionally, transcripts in the category “cellular component” were further assigned to 16 sub-categories; the three most represented sub-categories were “cell”, “cell part”, and “organelle”, which contained 1,600, 1,600, and 1,178 genes, respectively. Finally, transcripts in the category “molecular function” were mapped to 10 sub-categories; the sub-categories “binding” and “catalytic activity” were the most represented, containing over 3,600 and 2,800 genes, respectively. These results suggested that a large number of metabolic and cellular processes as well as binding and catalytic activities were occurring during the growth and development of *A. sibirica* seed kernels.

**Figure 9:**
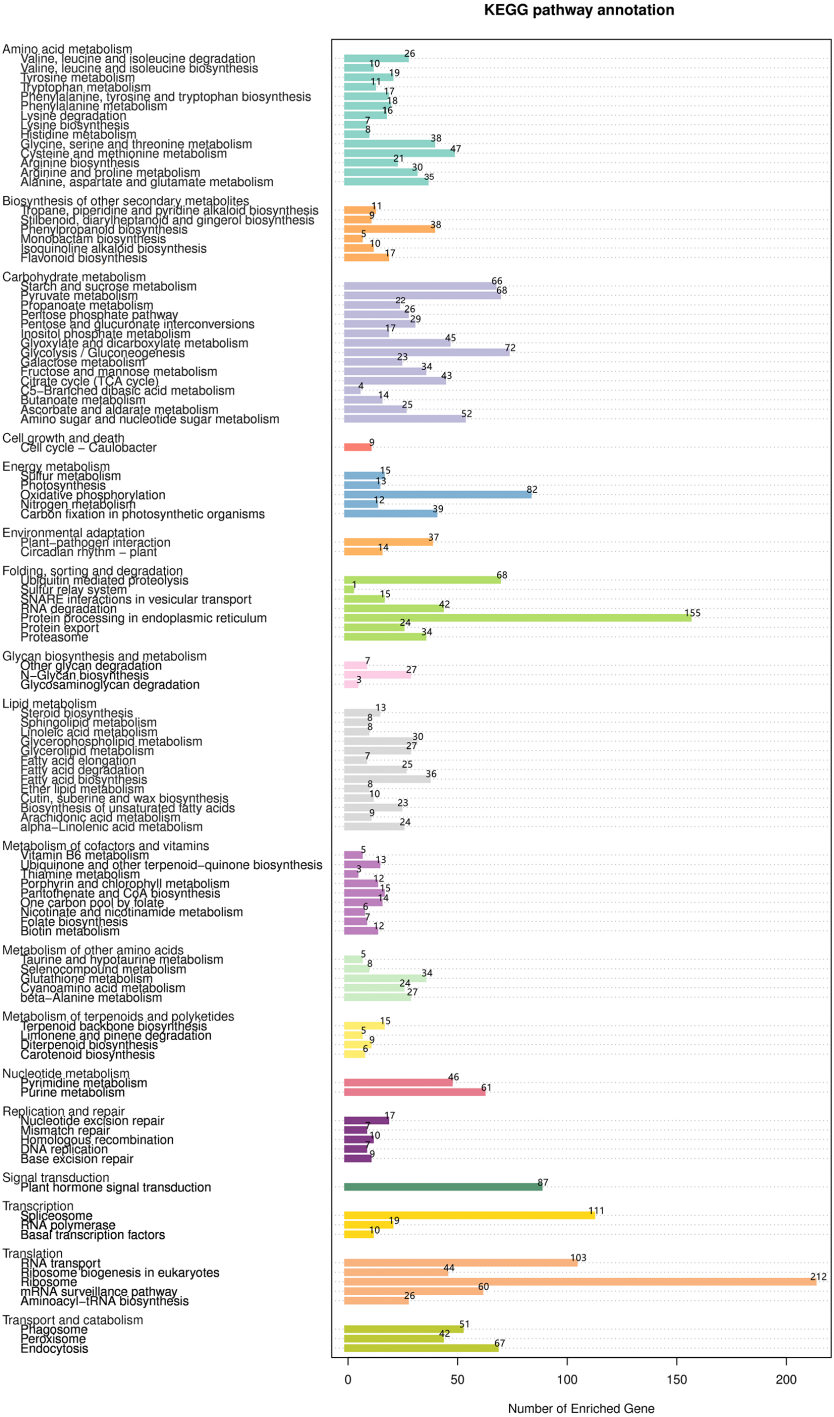
KEGG functional classification and pathway enrichment analysis of unigenes of *Armeniaca sibirica* seed kernels.

The KEGG database was used to evaluate completeness of transcriptome libraries and the effectiveness of transcriptome annotation. Transcripts were annotated into 18 KEGG categories (Fig. 9, Table S13). In the category “lipid metabolism”, 228 DEGs were annotated into 13 KEGG pathways; among these, the pathway “fatty acid biosynthesis” contained the largest number of DEGs (*n* = 36), followed by “glycerophospholipid metabolism” (*n* = 30), “glycerolipid metabolism” (*n* = 27), “fatty acid degradation” (*n* = 25), “alphalinolenic acid metabolism” (*n* = 24), and “biosynthesis of unsaturated fatty acids” (*n* = 23). Interestingly, “fatty acid elongation” contained only 7 DEGs.

### Hierarchical cluster analysis of DEGs associated with FAs accumulation

**Figure 10:**
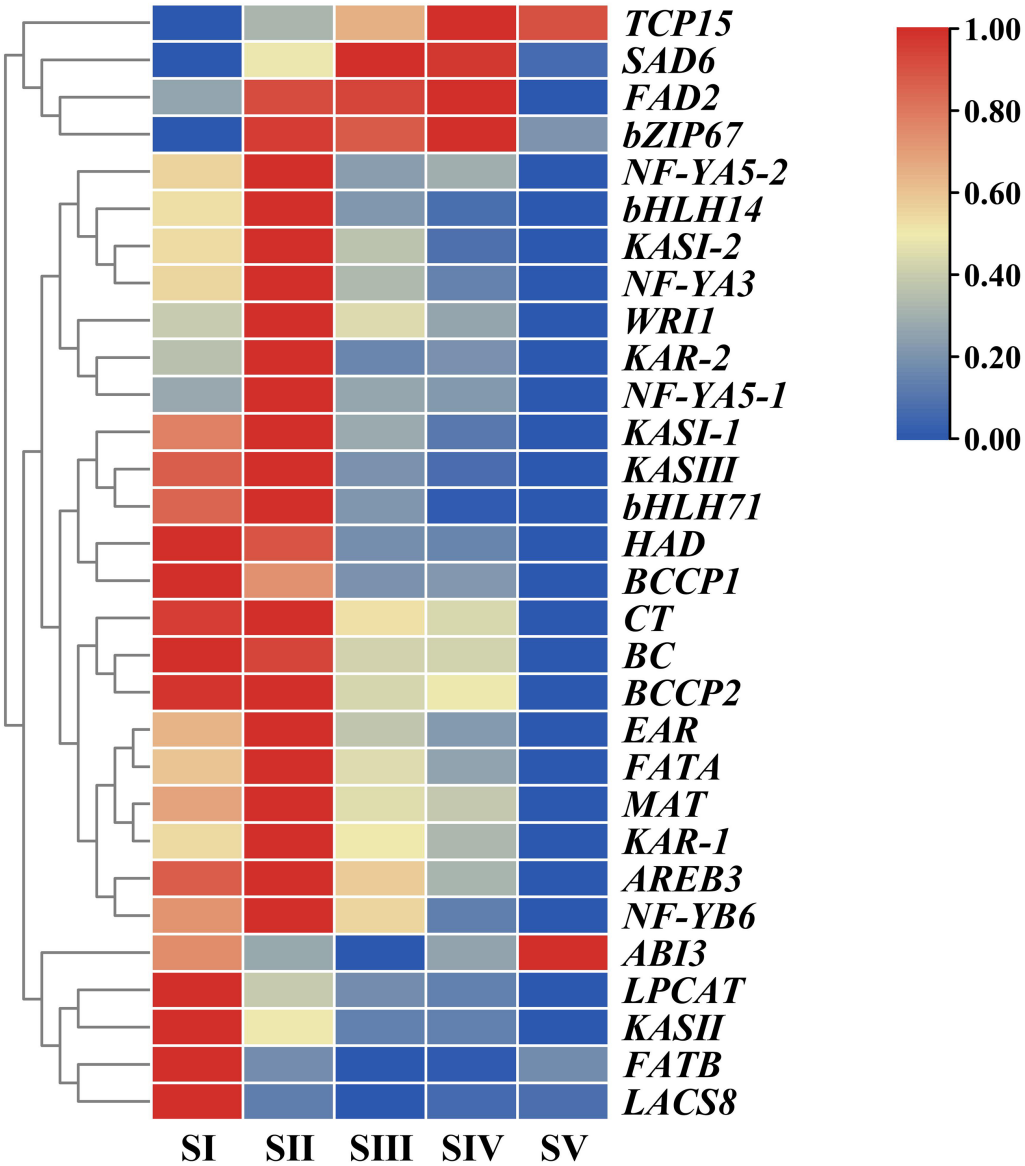
Cluster analysis of differentially expressed genes (DEGs) associated with FAs accumulation in *Armeniaca sibirica* seed kernels.

In order to unravel expression patterns of specific genes associated with FAs accumulation in *A. sibirica* seed kernels at different developmental stages, a hierarchical cluster analysis was conducted based on fragments per kilo base of transcript per million mapped fragments (FPKM) values of the selected unigenes (Fig. 10, Table S14). The number of upregulated genes was the highest at stage SII, followed by stage SI, and the lowest at stage SV; this indicated that genes related to FAs accumulation were mainly expressed at the early stages of *A. sibirica* kernel development. With the continuous increase in the production of intermediate products of reactions catalyzed by fatty acid synthase (FAS), the expression of genes related to FAs biosynthesis gradually decreased. However, expression of genes involved in the biosynthesis of oleic and linoleic acids (such as *SAD6* and *FAD2*) in stages SIII and SIV was remarkably high, and stabilized at stage SV. Collectively, these results were consistent with changes in FA composition of *A. sibirica* seed kernels at different developmental stages.

### Expression analysis of genes encoding key enzymes involved in FAs biosynthesis

**Figure 11:**
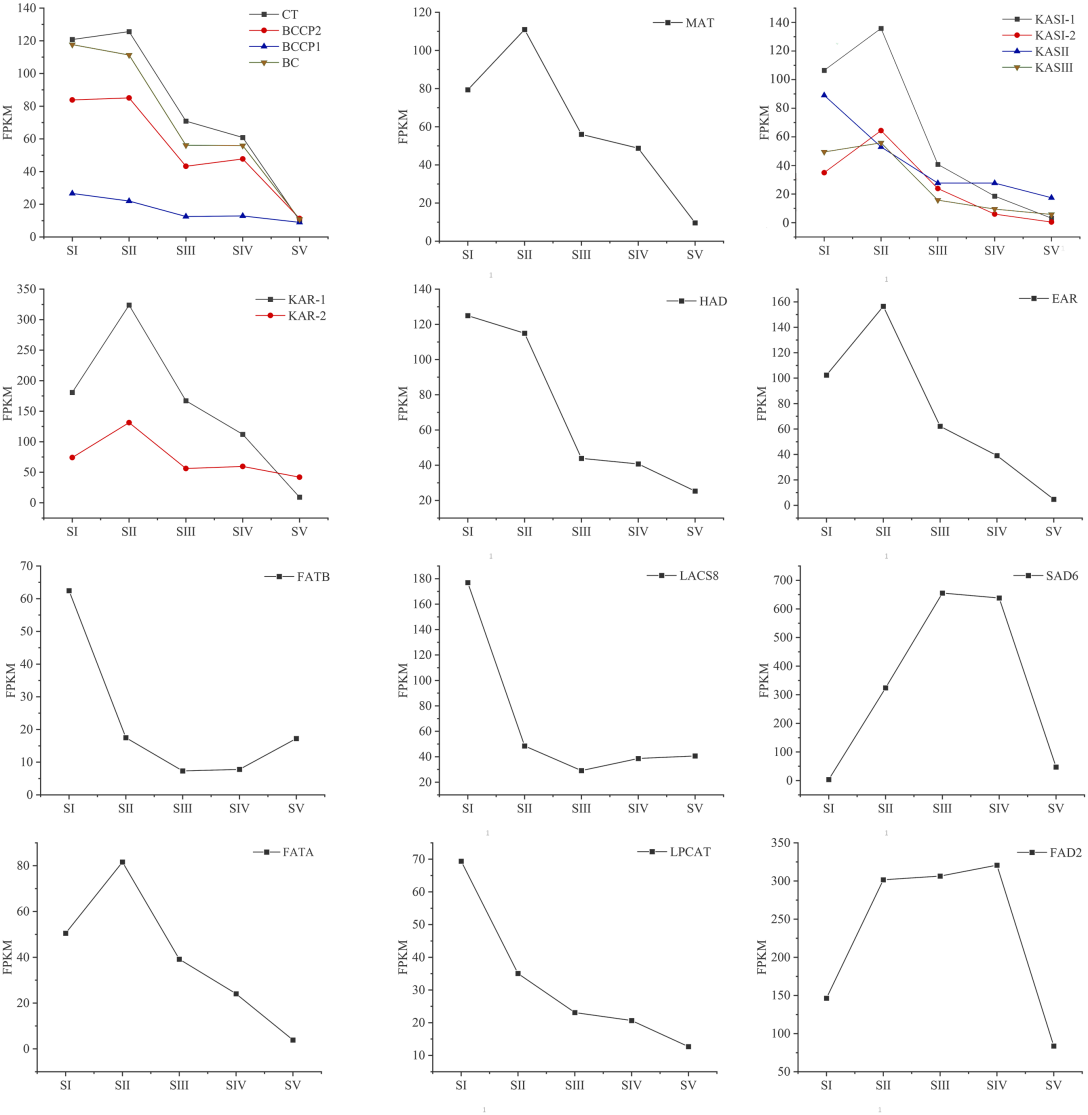
Temporal patterns of fragments per kilo base of transcript per million mapped fragments (FPKM) of genes encoding enzymes involved in the biosynthesis of fatty acids in *Armeniaca sibirica* seed kernels. Enzymatic abbreviations: BC, biotin carboxylase; BCCP, biotin carboxyl carrier protein; CT, carboxyl transferase; MAT, malonyl-CoA:ACP transferase; KAS I , 3-ketoacyl-ACP synthases I ; KASII, 3-ketoacyl-ACP synthases II; KASIII, 3-ketoacyl-ACP synthases III; KAR, ketoacyl-ACP reductase; HAD, hydroxyacyl-ACP dehydrase; EAR, enoyl-ACP reductase; FATB, fatty acyl-ACP thioesterase B; LACS8, long-chain acyl CoA synthetase 8; SAD6, Stearoyl-ACP desaturase 6; FATA, fatty acyl-ACP thioesterase A; LPCAT, acyl-CoA:lysophosphatidylcholine acyltransferase; FAD2, fatty acid desaturase 2.

Changes in the expression pattern of genes encoding key enzymes involved in FAs biosynthesis and metabolism in *A. sibirica* seed kernels at different developmental stages were analyzed (Fig. 11, Table S15). In the pathway “biosynthesis and metabolism” of FAs, the genes expressions of carboxyl transferase (CT), biotin carboxyl carrier protein (BCCP), biotin carboxylase (BC), malonyl-CoA: ACP transferase (MAT), 3-ketoacyl-ACP synthases isoform I and isoform III (KAS I and KASIII), ketoacyl-ACP reductase (KAR), hydroxyacyl-ACP dehydrase (HAD), enoyl-ACP reductase (EAR) and fatty acyl-ACP thioesterase A (FATA) increased from stages SI to SII, and then decreased from stages SII to SV. Interestingly, the expressions of a few genes in the above enzymes peaked at stage SI, and then decreased until stage SV. In UFAs biosynthesis, the gene expression of Stearoyl-ACP desaturase 6 (SAD6) gradually increased from stages SI to SIII, and maintained a high expression level from stages SIII to SIV, and decreased sharply from stages SIV to SV. Similarly, the gene expression of omega-6 FA desaturase (FAD2) increased significantly from stages SI to SII, and maintained a high expression level from stages SII to SIV, and then decreased at stage SV. Considering the negative regulation of FAs biosynthesis, the genes expressions of 3-ketoacyl-ACP synthases II (KASII), fatty acyl-ACP thioesterase B (FATB) and long-chain acyl-CoA synthetase 8 (LACS8) peaked at stage SI, then decreased from stages SI to SIII, and then slightly increased from stages SIII to SIV or SV. However, the gene expression of acyl-CoA: lysophosphatidylcholine acyltransferase (LPCAT) was highest at stages SI, then decreased from stages SI to SV.

### Differential expression of transcription factors involved in FAs biosynthesis

**Figure 12:**
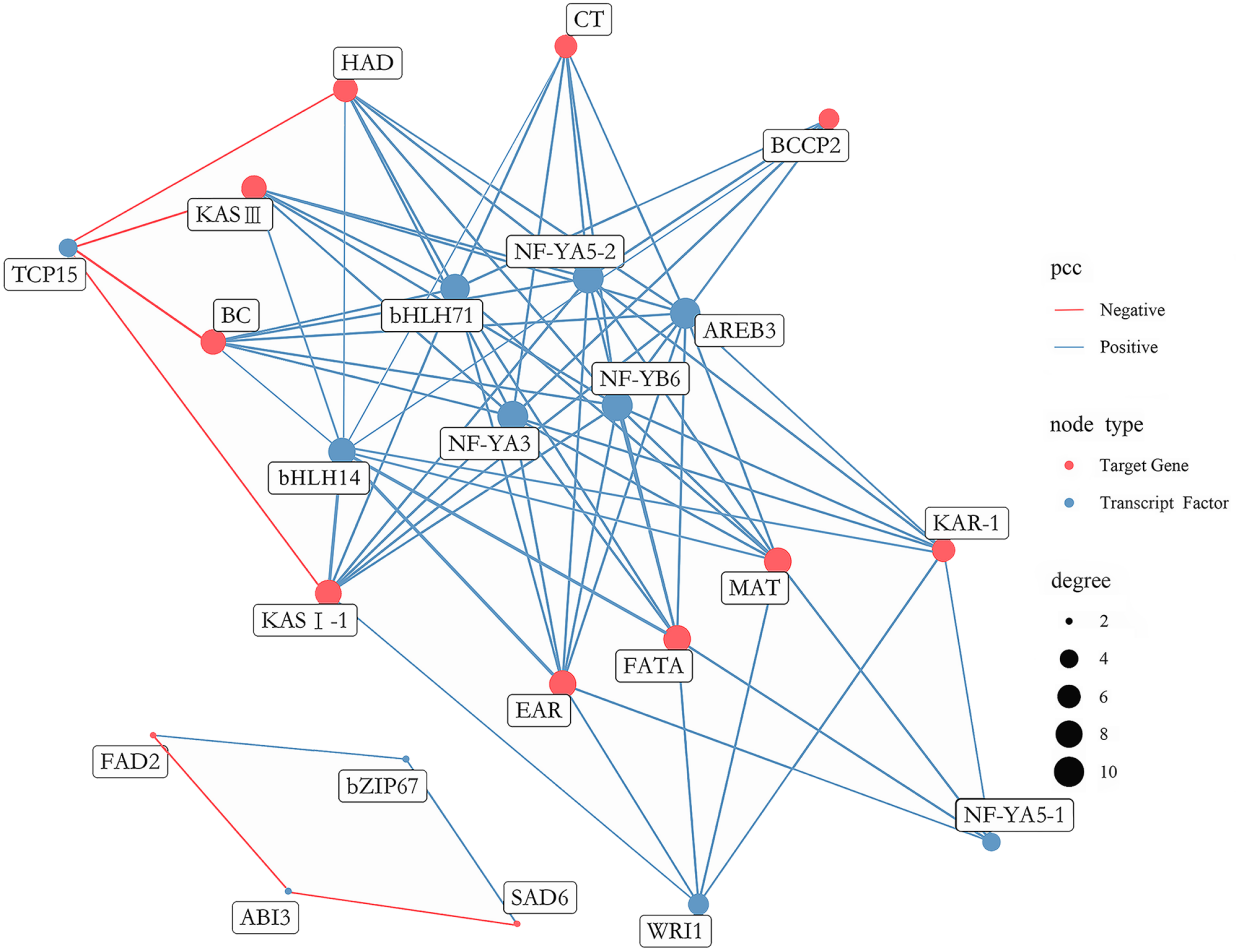
Co-expression network between lipid-related genes and transcription factors (TFs).

A total of 431 putative TFs were found among DEGs, which were further divided into 29 families (Table S16). To determine which TFs play a pivotal role in FAs biosynthesis in *A. sibirica* seed kernels, a gene co-expression network was constructed and analyzed considering differentially expressed TFs and the 12 most abundant DEGs encoding key enzymes involved in FAs biosynthesis (Fig. 12). TFs with high expression levels were included in 11 TFs identified by gene co-expression network analysis (Table S17). Collectively, nine and two TFs had significantly positive and negative co-expression, respectively, with genes coding key enzymes in FAs biosynthesis. The identified TFs belong to seven families, namely the basic leucine zipper (bZIP) family, B3 family, APETALA2 (AP2) family, basic helix-loop-helix (bHLH) family, nuclear factor-YA (NF-YA) family, nuclear factor-YB (NF-YB) family and Teosinte branched I, Cycloidea, Proliferating Cell Factors (TCP) family. Among the nine TFs related to positive regulation, bHLH14, bHLH71, NF-YA3, NF-YA5-2, NF-YB6 and AREB3 had the highest degrees of co-expression with lipid biosynthesis-related genes, indicating their potential participation in FAs biosynthesis. Among the two TFs related to negative regulation, TCP15 had the highest degree of co-expression with lipid biosynthesis-related genes, suggesting that these TFs might have an important role in FAs biosynthesis.

### Identification of lncRNAs involved in oil accumulation

LncRNAs are widely spread in several organisms, and it is known that some lncRNAs play important roles in multiple biological processes in plants (Shafiq, Li & Sun, 2016). Thus, we sought to identify lncRNAs in *A. sibirica* to uncover their potential relationships with FAs biosynthesis.

**Figure 13:**
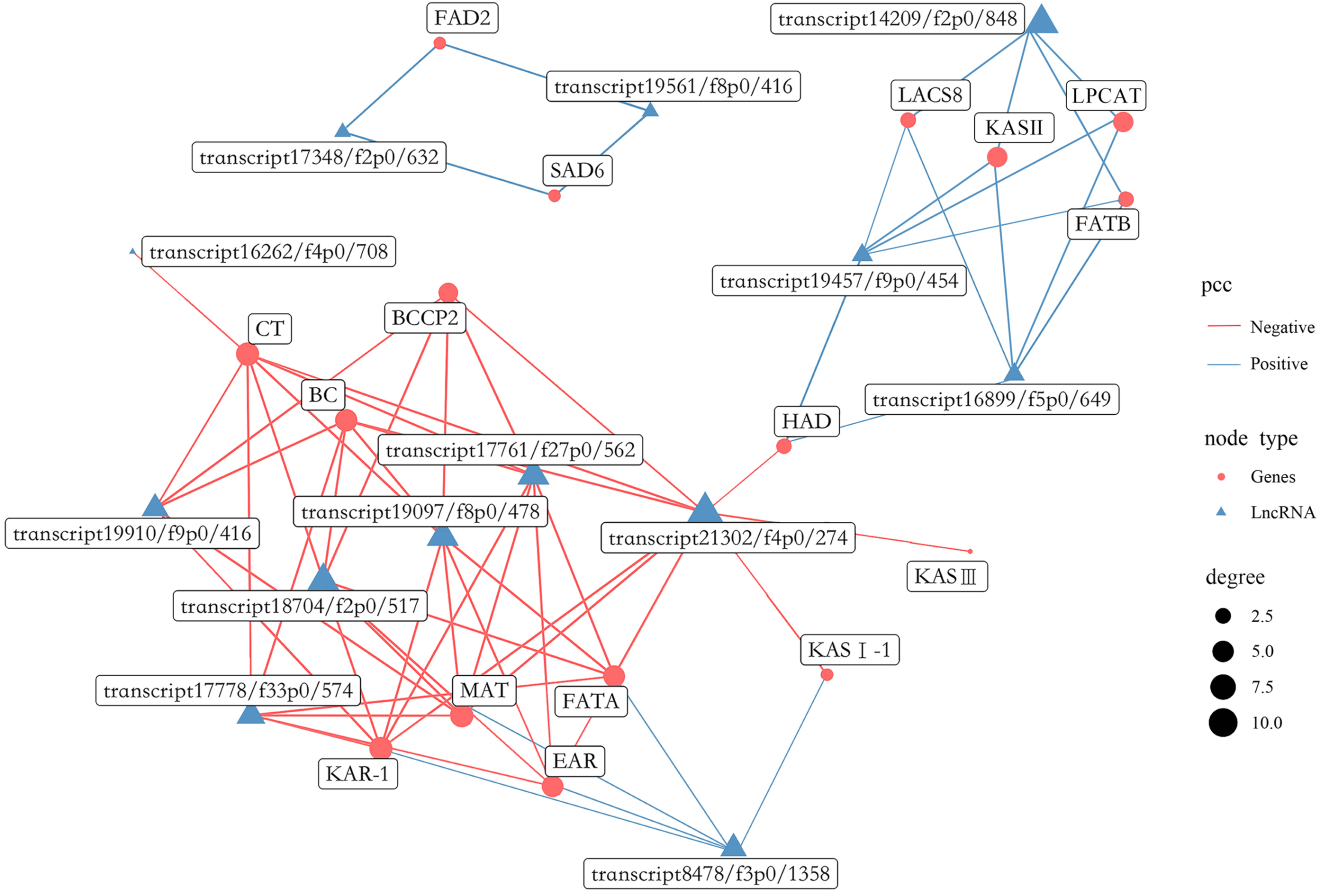
Gene co-expression network between lipid biosynthesis-related genes and lncRNAs in the transcriptome of *Armeniaca sibirica* seed kernels at different developmental stages.

A total of 1,738 lncRNAs were identified with an average length of 894 bp (Table S18). In the present study, certain lncRNAs were included among DEGs, which were then used to perform gene co-expression network analysis with the 16 most abundant DEGs related to FAs biosynthesis (Fig. 13, Table S19). Collectively, 13 lncRNAs were co-expressed with the 16 genes, including 6 related to positive regulation and seven related to negative regulation. In addition, eight lncRNAs had the highest degree of co-expression with nine DEGs related to lipid biosynthesis including *BC*, *BCCP2*, *CT*, *KAR-1*, *FATA*, *MAT, EAR, LPCAT* and *KAS II*.

### Validation of RNA-Seq data by qRT-PCR

**Figure 14:**
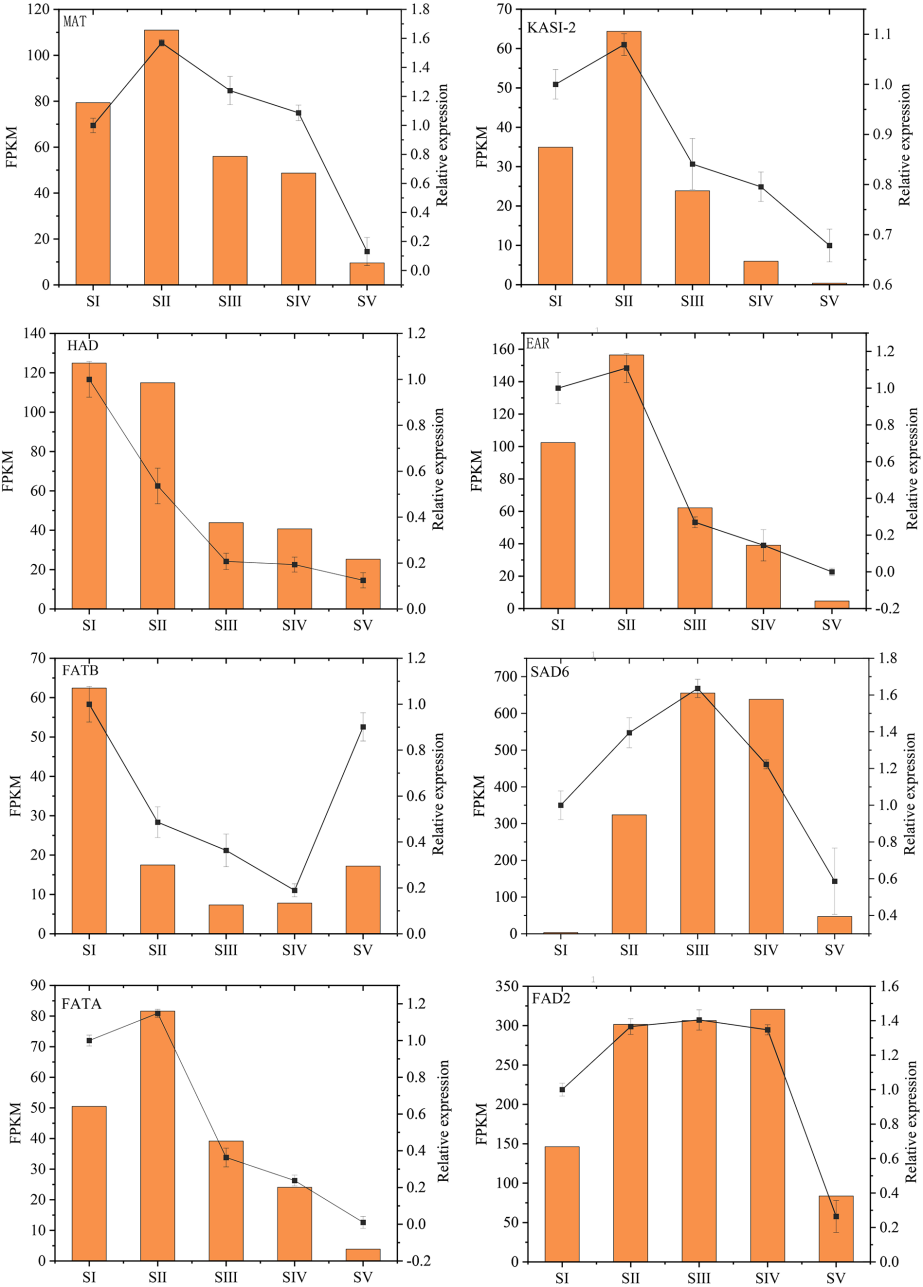
Validation of transcriptome data by qRT-PCR.

The relative expression levels of eight key genes involved in FAs accumulation in *A. sibirica* were randomly chosen and analyzed by qRT-PCR to validate RNA-Seq data (Fig. 14, Table S20). qRT-PCR and FPKM results were generally consistent with RNA-Seq findings. Thus, qRT-PCR results confirmed the validity of transcriptome findings.

## Discussion

**Figure 15:**
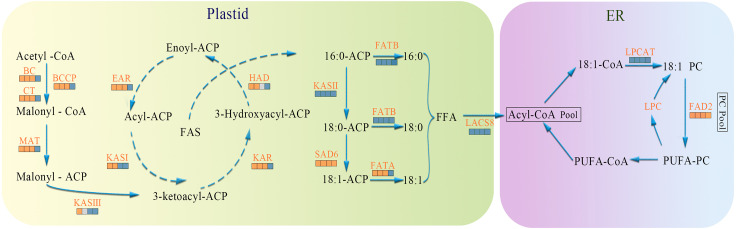
The temporal patterns for enzymes involved in fatty acids biosynthesis in *Armeniaca sibirica* seed kernels. The icons below each enzyme show the result of DESeq analysis,from left to right: SII:S I , SIII:S I , SIV:S I and SV:S I ; brown, up-regulation;gray, no significant difference;blue, down-regulation. Substrate abbreviations: acetyl-CoA, acetyl-coenzyme A; acyl-CoA, acyl-coenzyme A; malonyl-CoA, malonyl-coenzyme A; ACP, acyl carrier protein;FFA, free fatty acid;PC, phosphatidylcholine; PUFA, polyunsaturated fatty acids; Enzymatic abbreviations: BC, biotin carboxylase; BCCP, biotin carboxyl carrier protein; CT, carboxyl transferase; MAT, malonyl-CoA:ACP transferase; KAS I , 3-ketoacyl-ACP synthases I ; KASII, 3-ketoacyl-ACP synthases II; KASIII, 3-ketoacyl-ACP synthases III; KAR, ketoacyl-ACP reductase; HAD, hydroxyacyl-ACP dehydrase; EAR, enoyl-ACP reductase; FATA, fatty acyl-ACP thioesterase A; FATB, fatty acyl-ACP thioesterase B; SAD6, Stearoyl-ACP desaturase 6; LACS8, long-chain acyl CoA synthetase 8; LPCAT, acyl-CoA:lysophosphatidylcholine acyltransferase; FAD2, fatty acid desaturase 2.

In the present study, biosynthesis and regulation of FAs in *A. sibirica* seed kernels were explored at the transcriptomic level. Based on the findings of the present study and previous works, a scheme for the biosynthesis and regulation of FAs in *A. sibirica* seed kernels at different developmental stages is proposed, as well as the expression pattern of key genes identified in this study (Fig. 15).

In the FAs biosynthetic pathway, acetyl-CoA carboxylase (ACC) is a key rate-limiting enzyme that catalyzes the initial reaction in which acetyl-CoA is converted into malonyl-CoA, involving the biotin carboxyl carrier protein (BCCP), biotin carboxylase (BC), and the subunits of carboxyl transferase (CT) (Sasaki & Nagano, 2004). In the present study, the expression level of *BC* and *BCCP1* was the highest at stage SI, whereas the expression of *CT* and *BCCP2* was the highest at stage SII, followed by a decrease, reaching the lowest level at stage SV (Fig. 11). Thus, we speculated that the highest expression level of *acc* genes in early seed kernel development is important for the accumulation of FAs.

Before entering the FAs biosynthetic pathway, the malonyl group in malonyl-CoA produced by ACC needs to be transferred to ACP from CoA in a reaction catalyzed by malonyl-CoA: ACP transferase (MAT). Acetyl-CoA is used as the starting unit, and malonyl-ACP provides two-carbon units at each step of elongation. Malonyl-ACP enters a series of condensation, reduction, dehydration, and reduction reactions with acetyl-ACP (then acyl-ACP) in each cycle, being catalyzed by different enzymes. There are three 3-ketoacyl-ACP synthases (KAS) involved in the condensation reactions, which are KASIII, KAS I and KASII. Subsequent reduction, dehydration and reduction reactions are catalyzed by 3-ketoacyl-ACP reductase (KAR), hydroxyacyl-ACP dehydrase (HAD) and enoyl-ACP reductase (EAR) respectively (Ohirogge & Browse, 1995; Li-Beisson et al., 2013). In the present study, the expression levels of *MAT*, *KAS I*, *KASIII*, *KAR*, *HAD,* and *EAR* peaked at stage SI or SII, and then decreased the lowest value at SV (Fig. 11). Combined with the gradual increase in FAs synthesis from stages SI to SV (Fig. 1), it can be speculated that high expression levels of genes coding for key enzymes may be required in early stages of FAs synthesis, and subsequently, low expression levels could meet the need for precursors for FAs synthesis.

After seven elongation cycles, the production of saturated C16:0-ACP can be hydrolyzed to yield free C16:0 by fatty acyl-ACP thioesterase B (FATB), whereas C16:0-ACP is elongated by KASII to C18:0-ACP in the eighth cycle. Subsequently, C18:0-ACP can be hydrolyzed to yield free C18:0 by FATB or desaturated to 18:1-ACP by a Stearoyl-ACP desaturase 6 (SAD6). The resulting 18:1-ACP can either enter the glycerolipid pathway or be hydrolyzed by fatty acyl-ACP thioesterase A (FATA) for export from the plastid (Bates, Stymne & Ohlrogge, 2013). In the present study, *FATB* genes were persistently down-regulated, while *FATA* genes were notably up-regulated at stages SII, SIII and SIV (Fig. 15), suggesting that the resulting free C18:1 rather than C16:0 and C18:0 may be the main product of plastid FAs synthesis in *A. sibirica* developing seed kernels, which corroborates the data on FA composition (Table S3). Interestingly, the *SAD6* gene was continuously up-regulated at stages SII, SIII, SIV and SV (Fig. 15). In particular, the FPKM value at stages SIII and SIV was over 600 times higher compared to the FPKM value at stage SI (Fig. 11). Thus, it can be speculated that the upward trend in C18:1 (oleic acid) content paralleled temporal transcriptional patterns of *SAD6* gene in *A. sibirica* developing seed kernels (Fig. 3); thus, SAD6 could be considered a key enzyme for oleic acid formation in *A. sibirica* developing seed kernels.

Long-chain acyl groups are hydrolyzed by acyl-ACP thioesterases to release FAs which are ultimately activated to CoA esters by a long-chain acyl-CoA synthetase 8 (LACS8) and exported to the endoplasmic reticulum (ER) (Koo, Ohlrogge & Pollard, 2004). For polyunsaturated FAs synthesis, acyl-CoA: lysophosphatidylcholine acyltransferase (LPCAT) first catalyzes 18:1-CoA to generate 18:1 PC, which is then desaturated by omega-6 FA desaturase (FAD2) to 18:2 PC in phosphatidylcholine pool (PC pool). In our study, the *LACS8* gene had the highest expression level at stage SI, and the lowest expression level at stage SIII, followed by a slight increase at stages SIV and SV; in contrast, the expression of *LPCAT* gene continuously decreased from stages SI to SV (Fig. 11). The continuous down-regulation of *LACS8* and *LPCAT* genes (Fig. 15) may be related to the higher content of monounsaturated FAs compared to polyunsaturated fatty acids in *A. sibirica* seed kernels. In addition, the *FAD2* gene, as a key gene in FAs biosynthesis, maintained high expression levels from stages SII to SIV (Fig. 11), and its continuous up-regulation (Fig. 15) might also explain the high contents of linoleic acid (C18:2) (Fig. 3) in *A. sibirica* seed kernel oil. Differences in the contents of all types of FAs also indicated that certain genes coding for key enzymes, such as *KASII*, *SAD6*, *FATA*, *FATB*, *LACS8*, *LPCAT* and *FAD2*, might constitute a complex regulatory network in *A. sibirica* developing seed kernels.

The analysis of co-expression networks based on similarity of gene expression patterns is a powerful approach to accelerate the investigation of regulatory molecular mechanisms in important biological processes (Serin et al., 2016). Specifically, co-expression gene networks can help to discern causal relationships among genes. TFs can regulate the expression of a wide number of genes, playing key roles in metabolic networks (Grotewold, 2008). A study found that bHLH TF was shown to be involved in FA biosynthesis in high-yielding oil palm (Wong et al., 2017). AREB belongs to the bZIP family of TFs and has been reported to be a group of crucial regulators of seed lipid production in *Arabidopsis* (Mendes et al., 2013). Another study showed that NF-Y TF was related to PUFAs biosynthesis in the marine teleost *Siganus canaliculatus* (Dong et al., 2018). Several studies have indicated that WRI1 (AP2 family) and LEC1 (NF-Y family) were key TFs regulating oil biosynthesis in *Arabidopsis* (Baud & Lepiniec, 2010; To et al., 2012). In the present study, transcriptome analysis of FAs in *A. sibirica* seed kernels at different developmental stages revealed that the TFs bHLH14, bHLH71, NF-YA3, NF-YA5-2, NF-YB6 and AREB3 were highly expressed and established high degrees of interaction with lipid biosynthesis-related genes (Fig. 12). In addition, expression of TFs WRI1, NF-YA5-1 and TCP15 showed high correlation with lipid biosynthesis-related genes (Fig. 12). Thus, the findings of our study suggested that these TFs may play important roles in FAs biosynthesis in *A. sibirica* seed kernels.

More recently, studies indicated that plant lncRNAs are important regulators in multiple biological pathways, such as stress response to adversities, flower and fruit development (Huang et al., 2018; Sang et al., 2021; Li et al., 2022; Zhou et al., 2022). However, the role of lncRNAs in FAs synthesis in seeds has been poorly studied. In our study, 13 lncRNAs were found to be co-expressed with major lipid biosynthesis-related genes (Fig. 13). Further analysis revealed that the expression of eight lncRNAs, namely transcript 19910/f9p0/416, transcript 17778/f33p0/574, transcript 18704/f2p0/517, transcript 19097/f8p0/478, transcript 17761/f27p0/562, transcript 21302/f4p0/274, transcript 8478/f3p0/1358 and transcript 14209/f2p0/1848 were highly correlated with several important lipid biosynthesis-related genes (Fig. 13). Therefore, these 8 lncRNAs may have substantial functions in FAs biosynthesis in *A. sibirica* seed kernels. The co-expression of lncRNAs as positive and negative regulators may contribute to the dynamic balance of FAs biosynthesis in *A. sibirica* seed kernels.

## Conclusions

In the present study, biosynthesis and regulation of FAs in *A. sibirica* seed kernels at different stages of development were analyzed based on metabolome and transcriptome analyses. The genes encoding key enzymes and the expression profiles of known FAs biosynthesis-related genes in *A. sibirica* seed kernels at different stages of development were elucidated. In addition, certain TFs and lncRNAs potentially involved in FAs biosynthesis in *A. sibirica* seed kernels were described for the first time using co-expression gene networks. Collectively, the results discussed herein further clarify the regulatory mechanisms of FAs biosynthesis in developing seed kernels of *A. sibirica.* Future functional analysis of these newly identified candidate genes will further contribute to enlarging the current knowledge of FAs biosynthesis and regulation in *A. sibirica* seed kernels.

##  Supplemental Information

10.7717/peerj.14125/supp-1 Supplemental Information 1 Sequence information of primers for RT-qPCR

10.7717/peerj.14125/supp-2 Supplemental Information 2 The raw data for fatty acid content in *Armeniaca sibirica* seed kernel oil at different developmental stages

10.7717/peerj.14125/supp-3 Supplemental Information 3 The contents of various saturated fatty acids (SFAs) and unsaturated fatty acids (UFAs) in *Armeniaca sibirica* seed kernels at different developmental stages ( *μ*g/g)

10.7717/peerj.14125/supp-4 Supplemental Information 4 The content of total fatty acids in *Armeniaca sibirica* seed kernels at different developmental stages ( *μ*g/g)

10.7717/peerj.14125/supp-5 Supplemental Information 5 The relative proportion of saturated fatty acids (SFAs) and unsaturated fatty acids (UFAs) in *Armeniaca sibirica* seed kernels at different developmental stages

10.7717/peerj.14125/supp-6 Supplemental Information 6 The content of main unsaturated fatty acids in *Armeniaca sibirica* seed kernels at different developmental stages. (ug/g)

10.7717/peerj.14125/supp-7 Supplemental Information 7 The content of main saturated fatty acids in *Armeniaca sibirica* seed kernels at different developmental stages. (ug/g)

10.7717/peerj.14125/supp-8 Supplemental Information 8 Quality statistics for transcriptome sequencing (RNA-seq) data

10.7717/peerj.14125/supp-9 Supplemental Information 9 The summary statistics ofthe assembled transcripts and unigenes

10.7717/peerj.14125/supp-10 Supplemental Information 10 Detailed information of unigenes in the transcriptome of *Armeniaca sibirica* matching the 20 top species using BLAST against the Nr database

10.7717/peerj.14125/supp-11 Supplemental Information 11 Detailed information of KOG annotation of *Armeniaca sibirica* transcriptome

10.7717/peerj.14125/supp-12 Supplemental Information 12 Detailed information of GO annotation of *Armeniaca sibirica* transcriptome

10.7717/peerj.14125/supp-13 Supplemental Information 13 Detailed information of KEGG functional classification and pathway enrichment analysis of unigenes of *Armeniaca sibirica* seed kernels

10.7717/peerj.14125/supp-14 Supplemental Information 14 FPKM values of differentially expressed genes (DEGs) associated with FAs accumulation in *Armeniaca sibirica* seed kernels at different developmental stages

10.7717/peerj.14125/supp-15 Supplemental Information 15 FPKM of genes encoding enzymes involved in the biosynthesis of fatty acids in *Armeniaca sibirica* seed kernels at different developmental stages

10.7717/peerj.14125/supp-16 Supplemental Information 16 The identification result of transcription factor families and transcription factor numbers in *Armeniaca sibirica* seed kernels at different developmental stages

10.7717/peerj.14125/supp-17 Supplemental Information 17 Overview of differentially expressed transcription factors co-expressed with the major lipid-related genes in Armeniaca sibirica seed kernels

10.7717/peerj.14125/supp-18 Supplemental Information 18 LncRNA prediction results of CNCI software in *Armeniaca sibirica* seed kernels at different developmental stages

10.7717/peerj.14125/supp-19 Supplemental Information 19 Overview of the differentially expressed lncRNAs co-expressed with the major lipid-related genes in *Armeniaca sibirica* seed kernels

10.7717/peerj.14125/supp-20 Supplemental Information 20 The Ct value of sample

## Abbreviations

ABI3: abscisic acid insensitive 3
acetyl-CoA: acetyl-coenzyme A
ACP: acyl-carrier protein
ACC: acetyl-CoA carboxylase
AREB3: ABA-responsive bZIP-type transcription factor 3
BC: biotin carboxylase
BCCP: biotin carboxyl carrier protein
bHLH: basic helix-loop-helix
BLAST: Basic Local Alignment Search Tool
bZIP: basic leucine zipper
CT: carboxyl transferase
DEGs: differentially expressed genes
EAR: enoyl-ACP reductase
ER: endoplasmic reticulum
ERF: ethylene-responsive factor
FAD2: omega-6 FA desaturase
FATA: fatty acyl-ACP thioesterase A
FATB: fatty acyl-ACP thioesterase B
FAs: fatty acids
FAS: fatty acid synthases
FPKM: fragments per kilo base of transcript per million mapped fragments
GO: gene ontology
HAD: hydroxyacyl-ACP dehydrase
KAR: ketoacyl-ACP reductase
KAS I: 3-ketoacyl-ACP synthases I
KASII: 3-ketoacyl-ACP synthases II
KASIII: 3-ketoacyl-ACP synthases III
KEGG: Kyoto Encyclopedia of Genes and Genomes
KOG: euKaryotic Ortholog Groups
LACS8: long-chain acyl CoA synthetase 8
lncRNA: long non-coding RNA
LPCAT: acyl-CoA:lysophosphatidylcholine acyltransferase
MAT: malonyl-CoA:ACP transferase
MUFAs: monounsaturated fatty acids
NF-Y: nuclear factor-Y
Nr: NCBI Non-redundant protein
Nt: NCBI Nucleotide sequences
PC: phosphatidylcholine
PUFAs: polyunsaturated fatty acids
qRT-PCR: quantitative real-time PCR
RNA-Seq: RNA sequencing
SAD6: Stearoyl-ACP desaturase 6
SFAs: saturated fatty acids
TCP: Teosinte branched I, Cycloidea, Proliferating Cell Factors
TFs: transcription factors
UFAs: unsaturated fatty acids
WRI1: WRINKLED1

## Additional Information and Declarations

### DNA Deposition

The following information was supplied regarding the deposition of DNA sequences: The transcript sequence data is available at NCBI: PRJNA799670, PRJNA799941, PRJNA803801. The assembled data is available at NCBI: GJXS00000000.

### Data Availability

The following information was supplied regarding data availability: The raw data are available in the Supplementary Files.
