# Supplementary material for: Regulatory mechanisms of fatty acids biosynthesis in Armeniaca sibirica seed kernel oil at different developmental stages
Source: PeerJ. 2022 Oct 4;10:e14125. doi: 10.7717/peerj.14125 (PMC9541615; doi:10.7717/peerj.14125)
Supplement: Supplemental Information 1 [file peerj-10-14125-s001.docx]

Table S1 Sequence information of primers for RT-qPCR

| **Gene** | **Primer** | **Primer sequence（5’-3’）** |
| --- | --- | --- |
| MAT | MAT-F | TGTCACAAGTCTAGCCGCTG |
|  | MAT-R | TGAGCCCATCCTCAAAGCTG |
| KASI-2 | KASI-2-F | TATTATGGTAGTTGGTGGGAC |
|  | KASI-2-R | TATCGGTGCTCCTCTGTTC |
| HAD | HAD-F | CTCCTGCTCCCAGTTCGCAG |
|  | HAD-R | GGGGTCTCCTTGTTGGGAGC |
| EAR | EAR-F | CAGTCCATTGCCAGGATTGC |
|  | EAR-R | CCCACGCCTTAAACTGGATTC |
| FATB | FATB-F | TGGGTTAGTGCCTCTGGG |
|  | FATB-R | TTCCTGCTGTCCTCCTCC |
| SAD6 | SAD6-F | TCTCTGCCCCCTGAAAAAGTC |
|  | SAD6-R | AAAATACTCGTCCGGTAGCTCA |
| FATA | FATA-F | GGGAAGGGAGAATTGGTAC |
|  | FATA-R | TATTGTTAGGCTCTGGAAAG |
| FAD2 | FAD2-F | TGGCCAGTTTACTGGTTCGT |
|  | FAD2-R | TGTGTTAGAGTGGTGACGGC |
| 18SrRNA | 18SrRNA-F | AAACGGCTACCACATCCA |
|  | 18SrRNA-R | CACCAGACTTGCCCTCCA |
