# Supplementary material for: Regulatory mechanisms of fatty acids biosynthesis in Armeniaca sibirica seed kernel oil at different developmental stages
Source: PeerJ. 2022 Oct 4;10:e14125. doi: 10.7717/peerj.14125 (PMC9541615; doi:10.7717/peerj.14125)
Supplement: Supplemental Information 3 [file peerj-10-14125-s003.docx]

| Fatty acids | Developmental stages (Mean±SD) | | | | |
| --- | --- | --- | --- | --- | --- |
|  | SⅠ | SⅡ | SⅢ | SⅣ | SⅤ |
| C6:0 Caproic | 0.65±0.05 | 0.60±0.06 | 0 | 0 | 0 |
| C8:0 Caprylic | 0.35±0.04 | 0.37±0.04 | 0 | 0 | 0 |
| C10:0 Capric | 0.29±0.05 | 0.37±0.04 | 0.15±0.10 | 0.65±0.12 | 0.73±0.37 |
| C12:0 Lauric | 3.26±0.54 | 3.50±0.58 | 3.97±0.47 | 4.86±0.70 | 5.43±1.27 |
| C14:0 Myristic | 6.00±1.09 | 8.64±1.04 | 14.34±0.98 | 19.17±1.64 | 19.84±1.94 |
| C16:0 Palmitic | 761.33±72.97 | 1040.04±115.50 | 3475.13±342.53 | 4666.18±514.95 | 4940.87±480.25 |
| C17:0 Heptadecanoic | 5.23±0.52 | 8.93±1.47 | 19.54±2.65 | 30.54±4.50 | 34.40±5.32 |
| C18:0 Stearic | 569.30±54.07 | 635.46±76.77 | 1241.11±73.52 | 1406.88±157.45 | 1507.94±129.99 |
| C20:0 Arachidic | 9.55±0.94 | 19.23±2.88 | 51.01±3.57 | 62.97±6.00 | 73.96±12.52 |
| C21:0 Heneicosanoic | 0.99±0.15 | 2.06±0.46 | 0.14±0.19 | 0.47±0.50 | 0.89±0.81 |
| C22:0 Behenic | 4.00±0.43 | 6.58±0.95 | 11.95±0.82 | 14.65±2.09 | 18.68±3.03 |
| C23:0 Tricosanoic | 1.17±0.13 | 2.53±0.52 | 5.03±0.47 | 6.44±1.15 | 8.13±1.59 |
| C24:0 Lignoceric | 2.61±0.31 | 7.14±1.07 | 12.58±1.07 | 15.00±2.55 | 19.28±3.27 |
| **Total of saturated fatty acids(SFAs)** | **1364.73** | **1735.45** | **4834.95** | **6227.81** | **6630.15** |
| C16:1 Palmitoleic | 0.00 | 12.08±4.31 | 126.74±36.41 | 339.80±89.86 | 470.10±92.08 |
| C17:1 Heptadecenoic | 18.20±2.07 | 11.91±3.94 | 105.00±36.09 | 49.78±33.50 | 31.10±20.66 |
| C18:1 Oleic | 66.76±7.21 | 962.49±301.22 | 15769.26±2639.01 | 29284.24±4180.11 | 33554.45±3290.98 |
| C20:1 Gondoic | 2.31±0.36 | 9.44±2.32 | 45.03±4.48 | 79.14±13.69 | 98.78±17.96 |
| C22:1 Erucic | 4.44±1.04 | 4.55±0.70 | 9.18±1.92 | 8.47±1.59 | 6.89±0.88 |
| C18:2 Linoleic | 133.46±23.85 | 1785.13±399.46 | 12896.98±1434.34 | 19854.03±3222.91 | 21325.45±2471.35 |
| C18:3N6 γ-Linolenic | 0.00 | 0.00 | 0.72±1.77 | 7.69±3.40 | 8.46±3.11 |
| C18:3N3 α-Linolenic | 126.06±14.34 | 201.79±38.68 | 235.83±11.59 | 197.54±34.10 | 157.74±35.94 |
| C20:2 11,14-Eicosadienoic | 0.75±0.09 | 2.19±0.45 | 5.72±0.46 | 6.50±0.76 | 6.75±1.08 |
| C20:3 11,14,17-Eicosatrienoic | 0.71±0.07 | 0.74±0.15 | 3.49±0.64 | 3.12±0.52 | 3.05±0.52 |
| C20:5 Eicosapentaenoic | 0.22±0.13 | 0.35±0.03 | 0.00 | 0.00 | 0.00 |
| **Total of unsaturated fatty acids(UFAs)** | **352.95** | **2990.67** | **29197.95** | **49830.31** | **55662.77** |

**Table S3** The contents of various saturated fatty acids (SFAs) and unsaturated fatty acids (UFAs) in *Armeniaca sibirica* seed kernels at different developmental stages (μg/g)

Six biological replicates were performed for each developmental stage
