# Supplementary material for: Regulatory mechanisms of fatty acids biosynthesis in Armeniaca sibirica seed kernel oil at different developmental stages
Source: PeerJ. 2022 Oct 4;10:e14125. doi: 10.7717/peerj.14125 (PMC9541615; doi:10.7717/peerj.14125)
Supplement: Supplemental Information 4 [file peerj-10-14125-s004.docx]

**Table S4** The content of total fatty acids in *Armeniaca sibirica* seed kernels at different developmental stages (μg/g)

| replicates | Developmental stages | | | | |
| --- | --- | --- | --- | --- | --- |
|  | SⅠ | SⅡ | SⅢ | SⅣ | SⅤ |
| 1 | 1565.20 | 4233.29 | 32195.78 | 47815.07 | 60977.61 |
| 2 | 1887.28 | 4563.35 | 32320.94 | 49393.22 | 66825.20 |
| 3 | 1865.84 | 3386.16 | 28232.18 | 52063.32 | 71498.11 |
| 4 | 1533.08 | 5176.82 | 41432.57 | 62605.36 | 60038.99 |
| 5 | 1731.94 | 5895.69 | 34671.32 | 64815.32 | 54017.70 |
| 6 | 1722.59 | 5101.38 | 35344.62 | 59656.47 | 60400.06 |
| Mean±SD | 1717.65±147.15C | 4726.12±869.10C | 34032.90±4400.85B | 56058.13±7222.61A | 62292.94±6068.76A |

| Six biological replicates were performed for each developmental stage. Different capital letters indicate significant differences (*p* < 0.01). |
| --- |
