# Supplementary material for: Regulatory mechanisms of fatty acids biosynthesis in Armeniaca sibirica seed kernel oil at different developmental stages
Source: PeerJ. 2022 Oct 4;10:e14125. doi: 10.7717/peerj.14125 (PMC9541615; doi:10.7717/peerj.14125)
Supplement: Supplemental Information 5 [file peerj-10-14125-s005.docx]

**Table S5** The relative proportion of saturated fatty acids (SFAs) and unsaturated fatty acids (UFAs) in *Armeniaca sibirica* seed kernels at different developmental stages

| replicates | Developmental stages  (SFAs/T)% | | | | |
| --- | --- | --- | --- | --- | --- |
|  | SⅠ | SⅡ | SⅢ | SⅣ | SⅤ |
| 1 | 80.62 | 42.89 | 15.37 | 11.49 | 10.52 |
| 2 | 77.71 | 36.25 | 14.89 | 11.25 | 10.80 |
| 3 | 81.36 | 43.54 | 14.77 | 11.71 | 10.11 |
| 4 | 76.29 | 31.81 | 13.18 | 11.23 | 11.17 |
| 5 | 80.58 | 35.02 | 14.18 | 10.30 | 10.54 |
| 6 | 79.92 | 34.44 | 13.29 | 10.92 | 10.79 |
| Mean±SD | 79.41±1.98A | 37.33±4.79B | 14.28±0.89C | 11.15±0.49C | 10.65±0.36C |
| replicates | Developmental stages  (UFAs/T)% | | | | |
|  | SⅠ | SⅡ | SⅢ | SⅣ | SⅤ |
| 1 | 19.38 | 57.11 | 84.63 | 88.51 | 89.48 |
| 2 | 22.29 | 63.75 | 85.11 | 88.75 | 89.20 |
| 3 | 18.64 | 56.46 | 85.23 | 88.29 | 89.89 |
| 4 | 23.71 | 68.19 | 86.82 | 88.77 | 88.83 |
| 5 | 19.42 | 64.98 | 85.82 | 89.70 | 89.46 |
| 6 | 20.08 | 65.56 | 86.71 | 89.08 | 89.21 |
| Mean±SD | 20.59±1.98C | 62.67±4.79B | 85.72±0.89A | 88.85±0.49A | 89.35±0.36A |

Six biological replicates were performed for each developmental stage. Different capital letters indicate significant differences (*p* < 0.01).
