# Supplementary material for: Regulatory mechanisms of fatty acids biosynthesis in Armeniaca sibirica seed kernel oil at different developmental stages
Source: PeerJ. 2022 Oct 4;10:e14125. doi: 10.7717/peerj.14125 (PMC9541615; doi:10.7717/peerj.14125)
Supplement: Supplemental Information 6 [file peerj-10-14125-s006.docx]

**Table S6** The content of main unsaturated fatty acids in *Armeniaca sibirica* seed kernels at different developmental stages. (ug/g)

|  | replicates | | developmental stage | | | | | | | | |
| --- | --- | --- | --- | --- | --- | --- | --- | --- | --- | --- | --- |
|  |  |  | SⅠ | SⅡ | | SⅢ | | SⅣ | | SⅤ | |
| C18:1 Oleic | 1 | 65.94 | | | 679.60 | | 14600.06 | | 25324.82 | | 32931.53 |
|  | 2 | 79.94 | | | 914.46 | | 14506.45 | | 24392.91 | | 35833.99 |
|  | 3 | 64.42 | | | 542.75 | | 12213.08 | | 27077.60 | | 37716.05 |
|  | 4 | 60.53 | | | 1242.53 | | 19851.29 | | 33118.44 | | 32799.32 |
|  | 5 | 68.99 | | | 1252.48 | | 16116.48 | | 31869.50 | | 28034.44 |
|  | 6 | 60.72 | | | 1143.16 | | 17328.18 | | 33922.17 | | 34011.35 |
| Mean±SD | | 66.76±7.21C | | | 962.49±301.22C | | 15769.26±2639.01B | | 29284.24±4180.11A | | 33554.45±3290.98A |
| C18:2 Linoleic | 1 | 99.87 | | | 1481.16 | | 12177.66 | | 16421.06 | | 20910.90 |
|  | 2 | 162.09 | | | 1790.02 | | 12480.87 | | 18829.14 | | 22972.73 |
|  | 3 | 121.82 | | | 1176.06 | | 11344.30 | | 18240.29 | | 25524.43 |
|  | 4 | 155.95 | | | 2057.95 | | 15570.98 | | 21642.60 | | 19756.66 |
|  | 5 | 119.60 | | | 2273.36 | | 13036.03 | | 25468.64 | | 19616.23 |
|  | 6 | 141.42 | | | 1932.24 | | 12772.01 | | 18522.42 | | 19171.72 |
| Mean±SD | | 133.46±23.85C | | | 1785.13±399.46C | | 12896.98±1434.34B | | 19854.03±3222.91A | | 21325.45±2471.35A |
| Other Unsaturated fatty acids | 1 | 137.49 | | | 256.87 | | 469.81 | | 573.32 | | 717.59 |
|  | 2 | 178.59 | | | 204.50 | | 521.39 | | 613.70 | | 798.63 |
|  | 3 | 161.59 | | | 192.96 | | 503.62 | | 647.98 | | 1031.87 |
|  | 4 | 147.08 | | | 229.69 | | 547.67 | | 816.55 | | 774.67 |
|  | 5 | 147.72 | | | 305.04 | | 601.25 | | 803.59 | | 674.73 |
|  | 6 | 143.72 | | | 269.25 | | 546.51 | | 697.14 | | 699.78 |
| Mean±SD | | 152.70±14.95C | | | 243.05±42.18C | | 531.71±44.80B | | 692.05±100.14A | | 782.88±130.52A |

Six biological replicates were performed for each developmental stage. Different capital letters indicate significant differences (*p* < 0.01).
