# Supplementary material for: Regulatory mechanisms of fatty acids biosynthesis in Armeniaca sibirica seed kernel oil at different developmental stages
Source: PeerJ. 2022 Oct 4;10:e14125. doi: 10.7717/peerj.14125 (PMC9541615; doi:10.7717/peerj.14125)
Supplement: Supplemental Information 7 [file peerj-10-14125-s007.docx]

Table S7 The content of main saturated fatty acids in *Armeniaca sibirica* seed kernels at different developmental stages (ug/g)

|  | replicates | | developmental stage | | | | | | | | |
| --- | --- | --- | --- | --- | --- | --- | --- | --- | --- | --- | --- |
|  |  |  | SⅠ | SⅡ | | SⅢ | | SⅣ | | SⅤ | |
| C16:0 Palmitic | 1 | 697.02 | | | 1080.16 | | 3534.91 | | 4054.82 | | 4825.10 |
|  | 2 | 813.07 | | | 1005.01 | | 3422.28 | | 4163.51 | | 5391.15 |
|  | 3 | 845.67 | | | 870.06 | | 2950.12 | | 4540.95 | | 5556.12 |
|  | 4 | 652.88 | | | 1003.81 | | 4005.67 | | 5262.79 | | 4949.80 |
|  | 5 | 791.08 | | | 1222.55 | | 3576.34 | | 5233.76 | | 4239.17 |
|  | 6 | 768.25 | | | 1058.68 | | 3361.46 | | 4741.26 | | 4683.90 |
| Mean±SD | | 761.33±72.97C | | | 1040.04±115.50C | | 3475.13±342.53B | | 4666.18±514.95A | | 4940.87±480.25A |
| C18:0 Stearic | 1 | 534.54 | | | 672.98 | | 1287.63 | | 1302.10 | | 1434.54 |
|  | 2 | 615.55 | | | 596.43 | | 1275.13 | | 1262.65 | | 1629.75 |
|  | 3 | 636.24 | | | 554.17 | | 1117.65 | | 1405.18 | | 1460.76 |
|  | 4 | 486.99 | | | 584.49 | | 1327.73 | | 1594.28 | | 1552.12 |
|  | 5 | 569.14 | | | 767.10 | | 1219.15 | | 1272.64 | | 1313.42 |
|  | 6 | 573.37 | | | 637.57 | | 1219.35 | | 1604.43 | | 1657.07 |
| Mean±SD | | 569.30±54.07C | | | 635.46±76.77C | | 1241.11±73.52B | | 1406.88±157.45AB | | 1507.94±129.99A |
| Other saturated fatty acids | 1 | 30.34 | | | 62.52 | | 125.70 | | 138.94 | | 157.94 |
|  | 2 | 38.03 | | | 52.93 | | 114.82 | | 131.31 | | 198.95 |
|  | 3 | 36.11 | | | 50.17 | | 103.41 | | 151.32 | | 208.88 |
|  | 4 | 29.65 | | | 58.36 | | 129.23 | | 170.70 | | 206.42 |
|  | 5 | 35.41 | | | 75.17 | | 122.07 | | 167.19 | | 139.71 |
|  | 6 | 35.10 | | | 60.48 | | 117.11 | | 169.03 | | 176.23 |
| Mean±SD | | 34.11±3.35E | | | 59.94±8.79D | | 118.72±9.19C | | 154.75±16.88B | | 181.35±28.30A |

Six biological replicates were performed for each developmental stage. Different capital letters indicate significant differences (*p* < 0.01).
