# Supplementary material for: Regulatory mechanisms of fatty acids biosynthesis in Armeniaca sibirica seed kernel oil at different developmental stages
Source: PeerJ. 2022 Oct 4;10:e14125. doi: 10.7717/peerj.14125 (PMC9541615; doi:10.7717/peerj.14125)
Supplement: Supplemental Information 8 [file peerj-10-14125-s008.docx]

**Table S8 Quality statistics for transcriptome sequencing (RNA-seq) data**

| Library ID | Sample name | Raw reads | Clean reads | Clean reads rate (%) | Clean bases  (Gb) | Error rate(%) | Q30(%) | GC content(%) | Total mapped reads | Total mapping reads percentage (%) |
| --- | --- | --- | --- | --- | --- | --- | --- | --- | --- | --- |
| FRAS19H001826-1a | SⅠ_1 | 51967504 | 51273990 | 98.67 | 7.69 | 0.03 | 92.34 | 45.48 | 35650514 | 69.53 |
| FRAS19H001826-2a | SⅠ_2 | 62777018 | 61828066 | 98.49 | 9.27 | 0.03 | 92.56 | 45.48 | 43309428 | 70.05 |
| FRAS19H001826-3a | SⅠ_3 | 48927514 | 48352576 | 98.82 | 7.25 | 0.03 | 92.68 | 45.56 | 33763926 | 69.83 |
| FRAS19H001827-1a | SⅡ_1 | 60381096 | 59547586 | 98.62 | 8.93 | 0.03 | 93.52 | 46.23 | 48128714 | 80.82 |
| FRAS19H001827-2a | SⅡ_2 | 53238592 | 52603528 | 98.81 | 7.89 | 0.03 | 93.57 | 46.29 | 42740436 | 81.25 |
| FRAS19H001827-3a | SⅡ_3 | 53383128 | 52712340 | 98.74 | 7.91 | 0.03 | 93.21 | 46.44 | 43175824 | 81.91 |
| FRAS19H001828-1a | SⅢ_1 | 43696778 | 43224714 | 98.92 | 6.48 | 0.03 | 91.86 | 48.40 | 37043388 | 85.70 |
| FRAS19H001828-2a | SⅢ_2 | 50820196 | 50233858 | 98.85 | 7.54 | 0.03 | 93.54 | 48.49 | 43164404 | 85.93 |
| FRAS19H001828-3a | SⅢ_3 | 50444398 | 49649158 | 98.42 | 7.45 | 0.03 | 93.41 | 48.33 | 42630936 | 85.86 |
| FRAS19H001829-1a | SⅣ_1 | 57951266 | 57052910 | 98.45 | 8.56 | 0.03 | 93.44 | 49.25 | 49246098 | 86.32 |
| FRAS19H001829-2a | SⅣ_2 | 46841164 | 46259588 | 98.76 | 6.94 | 0.03 | 93.69 | 49.48 | 40014352 | 86.50 |
| FRAS19H001829-3a | SⅣ_3 | 54205690 | 53497110 | 98.69 | 8.02 | 0.03 | 93.59 | 49.57 | 46224054 | 86.40 |
| FRAS19H001830-1a | SⅤ_1 | 46300140 | 45602250 | 98.49 | 6.84 | 0.03 | 92.74 | 49.19 | 38307884 | 84.00 |
| FRAS19H001830-2a | SⅤ_2 | 49130890 | 48460412 | 98.64 | 7.27 | 0.03 | 93.52 | 49.17 | 40649660 | 83.88 |
| FRAS19H001830-3a | SⅤ_3 | 46887132 | 46183940 | 98.50 | 6.93 | 0.03 | 93.78 | 49.28 | 38994926 | 84.43 |

Q30: The percentage of bases with Phred value > 30 to the total bases, where Phred=-10Log_10_e.
