# Supplementary material for: Regulatory mechanisms of fatty acids biosynthesis in Armeniaca sibirica seed kernel oil at different developmental stages
Source: PeerJ. 2022 Oct 4;10:e14125. doi: 10.7717/peerj.14125 (PMC9541615; doi:10.7717/peerj.14125)
Supplement: Supplemental Information 9 [file peerj-10-14125-s009.docx]

**Table S9 The summary statistics of the assembled transcripts and unigenes**

|  | Total_nucleotides | Total_number | Average length(bp) | Min length(bp) | Max length(bp) | N50(bp) |
| --- | --- | --- | --- | --- | --- | --- |
| Transcript | 26553499 | 21786 | 1219 | 57 | 4626 | 1546 |
| Unigene | 14580056 | 10093 | 1445 | 61 | 4626 | 1715 |
